# Synthesis and Investigation of Anti-Inflammatory Activity of New Thiourea Derivatives of Naproxen

**DOI:** 10.3390/ph16050666

**Published:** 2023-04-28

**Authors:** Nikola Nedeljković, Vladimir Dobričić, Jelena Bošković, Marina Vesović, Jovana Bradić, Marijana Anđić, Aleksandar Kočović, Nevena Jeremić, Jovana Novaković, Vladimir Jakovljević, Zorica Vujić, Miloš Nikolić

**Affiliations:** 1 Department of Pharmacy, Faculty of Medical Sciences, University of Kragujevac, Svetozara Markovica 69, 34000 Kragujevac, Serbia; 2 Department of Pharmaceutical Chemistry, University of Belgrade-Faculty of Pharmacy, Vojvode Stepe 450, 11221 Belgrade, Serbia; 3 Center of Excellence for Redox Balance Research in Cardiovascular and Metabolic Disorders, Svetozara Markovica 69, 34000 Kragujevac, Serbia; 4 1st Moscow State Medical, University IM Sechenov, Trubetskaya 8/2, 119991 Moscow, Russia; 5 Department of Physiology, Faculty of Medical Sciences, University of Kragujevac, Svetozara Markovica 69, 34000 Kragujevac, Serbia; 6 Department of Human Pathology, 1st Moscow State Medical, University IM Sechenov, Trubetskaya 8/2, 119991 Moscow, Russia

**Keywords:** naproxen, thiourea, carrageenan, paw edema, anti-inflammatory activity, COX-2, 5-LOX, molecular docking, FRED

## Abstract

The aim of the study was a synthesis and investigation of the dose-dependent anti-inflammatory effect of new thiourea derivatives of naproxen with selected aromatic amines and esters of aromatic amino acids. The results of the in vivo study indicate that derivatives of *m*-anisidine (**4**) and *N*-methyl tryptophan methyl ester (**7**) showed the most potent anti-inflammatory activity four hours after injection of carrageenan, with the percentage of inhibition of 54.01% and 54.12%, respectively. In vitro assays of COX-2 inhibition demonstrated that none of the tested compounds achieved 50% inhibition at concentrations lower than 100 µM. On the other hand, the aromatic amine derivatives (**1**–**5**) accomplished significant inhibition of 5-LOX, and the lowest IC_50_ value was observed for compound **4** (0.30 μM). High anti-edematous activity of compound **4** in the rat paw edema model, together with potent inhibition of 5-LOX, highlight this compound as a promising anti-inflammatory agent.

## 1. Introduction

Inflammation is defined as a local or systemic response to tissue damage or different stimuli, such as biological, chemical, physical, and thermal factors [1]. Increased inflammatory response and elevated concentration of proinflammatory markers, such as cytokines and inflammatory chemokines, introduce the organism into a state of hyperinflammation [2]. Hyperinflammation in combination with reactive oxygen species (ROS) produced by oxidative stress is an important factor contributing to the development of various diseases, such as arthritis, cardiovascular disease, cancer, diabetes, etc. [3,4]. Arachidonic acid (AA) is a polyunsaturated ω-6 fatty acid that can be metabolized through various catabolic pathways. The two most important metabolic pathways of arachidonic acid are catalyzed by the enzymes cyclooxygenase (COX) and 5-lipoxygenase (5-LOX) [5]. Two isoforms of COX enzyme are COX-1, which is constitutively expressed, and COX-2, which is induced by inflammatory factors, hormones, and growth factors. Two steps in the biosynthesis of leukotrienes are catalyzed by 5-LOX: the biotransformation of AA to the intermediate 5-hydroperoxyeicosatetraenoic acid and its further transformation to leukotrienes. On the other hand, 5-LOX is also involved in the catalysis of lipid peroxidation by producing lipid peroxides. These peroxides promote membrane rupture and cell death, leading to the inflammatory response through the release of damage-associated molecular patterns (DAMPs) [6]. Inhibitors of this enzyme are being developed for the treatment of numerous human diseases and pathological conditions, such as asthma, allergic reactions, atherosclerosis, diabetes, and Alzheimer’s disease [7,8,9,10,11,12]. There is strong evidence that increased 5-LOX activity is associated with a process of neuroinflammation that causes memory deficits, while decreased enzyme expression promotes cognitive recovery [12].

Non-steroidal anti-inflammatory drugs (NSAIDs) are effective in the treatment of pain and inflammation and represent the most popular group of over-the-counter medications worldwide [13]. The main mode of NSAID action is the inhibition of cyclooxygenase enzymes, leading to the reduction in prostanoid biosynthesis. Long-term use of NSAIDs may induce gastrointestinal (GI) complications related to nonselective inhibition of both isoforms, whereas selective inhibition of COX-2 can lead to cardiovascular side effects [14,15].

Since FDA approval in the late 20th century, naproxen became one of the leading NSAIDs [16]. The bicyclic propionic acid derivative *S*-naproxen exhibits analgesic and anti-inflammatory activity through non-selective reversible inhibition of cyclooxygenases, but with approximately five times greater selectivity for COX-1 [17]. As a consequence of this selectivity profile, the production of physiologically active prostaglandins in the gastric mucosa is impaired, resulting in GI toxicity [18,19]. In addition, the free carboxyl group of naproxen may have a local erosive effect on the gastric mucosa due to its acidity. Masking this functional group with other biologically active organic motifs could be a promising strategy for the synthesis of new compounds that are more potent and less ulcerotoxic anti-inflammatory agents [20,21,22,23]. This structural modification could reduce the ability of naproxen to inhibit the COX-1 and retain the inhibitory affinity for COX-2 [24].

In recent years, the synthesis and in vivo biological testing of various compounds based on the naproxen scaffold attracted considerable attention among medicinal chemists. Naproxen derivatives with substituted 1,2,4-triazole rings exhibited significant anti-inflammatory activity without concomitant ulcerogenic activity [25]. Various amide compounds of naproxen, including glycolamides [26], carboxamides [27], and amino acid derivatives [28], showed pronounced anti-inflammatory activity in the carrageenan-induced paw edema model of acute inflammation in comparison to naproxen.

The thiourea moiety was described as an important pharmacophore in a variety of pharmacologically active compounds [29], including anti-inflammatory [30], anticancer [31], and antimicrobial agents [32]. On the other hand, the thiourea derivatives of naproxen showed pronounced anti-inflammatory activity in some previous studies. Thus, thiourea derivatives of naproxen with aminopyridines [33], 4-chloroaniline [34], and amino acids [35] demonstrated a higher percentage of the paw edema reduction in comparison to naproxen and showed insignificant ulcerogenic effects.

The concept of exploring dual COX-2 and 5-LOX inhibitors is applied with the aim to develop new drugs that have less pronounced side effects and retain a high level of anti-inflammatory activity. Two new molecules are currently in the third phase of clinical trials: licofelone, a potential drug for the treatment of osteoarthritis, and darbufelone, a potential drug for the treatment of rheumatoid arthritis [36,37].

The molecular docking is widely used within structure-based drug design and represents an in silico approach to identify new lead molecules and drug candidates [38,39]. With the aim to identify small molecule inhibitors against specific molecular targets, molecular docking analysis can provide insight into the ligand–receptor interactions, enabling virtual screening of numerous synthetic [40] and naturally occurring compounds [41,42].

In the present study, the dose-dependent anti-inflammatory effect of newly synthesized thiourea derivatives of naproxen was investigated using the in vivo carrageenan-induced paw edema model of acute inflammation. In vitro assays of COX-2 and 5-LOX enzyme inhibition and in silico investigation of the synthesized compounds–enzymes (COX-2 and 5-LOX) interactions were also performed.

## 2. Results and Discussion

### 2.1. General Procedure for the Synthesis of Thiourea Derivatives of Naproxen

In the present study, we synthesized thiourea derivatives of naproxen with selected aromatic amines and aromatic amino acid esters, which were not previously synthesized and characterized in the literature. This synthetic approach based on chemical modification of naproxen scaffold was used with the aim to obtain molecules with more potent anti-inflammatory activity and improved safety.

**Scheme 1:**
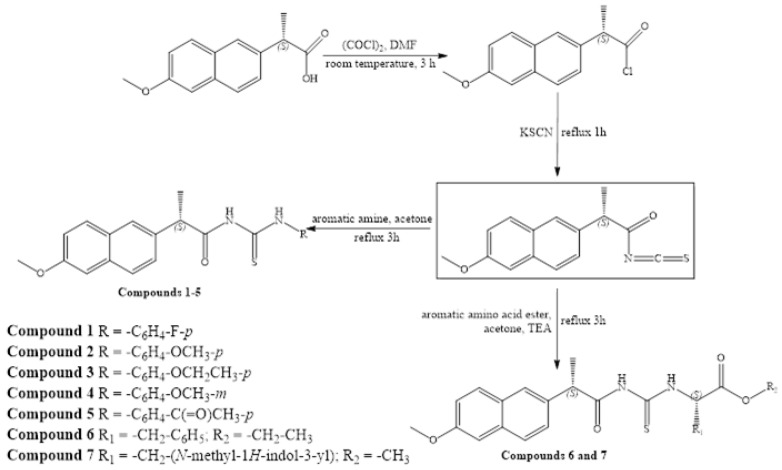
Synthesis of tested compounds.

The synthesis was carried out according to previously described procedure for similar compounds [35,43], using S-naproxen as a starting compound, which in the reaction with oxalyl chloride is converted into naproxenoyl chloride. Naproxenoyl chloride in the presence of potassium thiocyanate reacted with aromatic amines (compounds **1**–**5**) or esters of aromatic amino acids (compounds **6** and **7**), whereby the investigated compounds were obtained (Scheme 1). Our strategy for the synthesis of naproxen derivatives relies on the assumption that conversion of the carboxyl group to thiourea moiety using lipophilic and sterically bulky aromatic amines and esters of aromatic amino acids would produce derivatives with significant anti-inflammatory activity and better gastric tolerability in comparison to naproxen [20,21,22,23].

In the ^1^H NMR spectrum of the *p*-fluoroaniline derivative (compound **1**), the CH_3_ group from the naproxen scaffold (C**H_3_**_Nap_) gave doublet at 1.51 ppm. The methoxy group (O**C**H_3Nap_) gave a signal at 3.87 ppm, while the signal of hydrogen at the chiral carbon (**C**H_Nap_) appeared at 4.22 ppm. Aromatic protons gave signals in the range from 7.16 to 7.85 ppm. This derivative exhibited two signals at 11.72 and 12.31 ppm, attributed to N**H**CS and CSN**H**, respectively. In the ^13^C NMR spectrum, **C**H_3Nap_, **C**H_Nap_, and O**C**H_3Nap_ atoms gave signals at 17.45, 44.35, and 54.59 ppm, respectively. Signals of the naphthalene and benzene nuclei were between 105.12 and 134.66 ppm (doublets that belong to carbons in the 4-fluorophenyl ring were 114.71 (CF**C**HCHCH), 126.31 (CFCH**C**HCH), 133.51 (CFCHCH**C**H), and 159.40 (**C**FCHCHCH). Two close carbonyl and thiocarbonyl signals originating from the thioureido group were at 175.46 and 178.97 ppm. The NMR spectra of all synthesized compounds are presented in Supplementary Materials. The HRMS spectrum showed the adduct, with sodium [M+Na]^+^ having m/z ratio 405.10432 (calculated value: 405.10435; error: 0.07 ppm).

### 2.2. Acute Oral Toxicity Evaluation

With the aim to define the safety profile of newly synthesized compounds, their toxicity on vital organs were examined.

**Figure 1:**
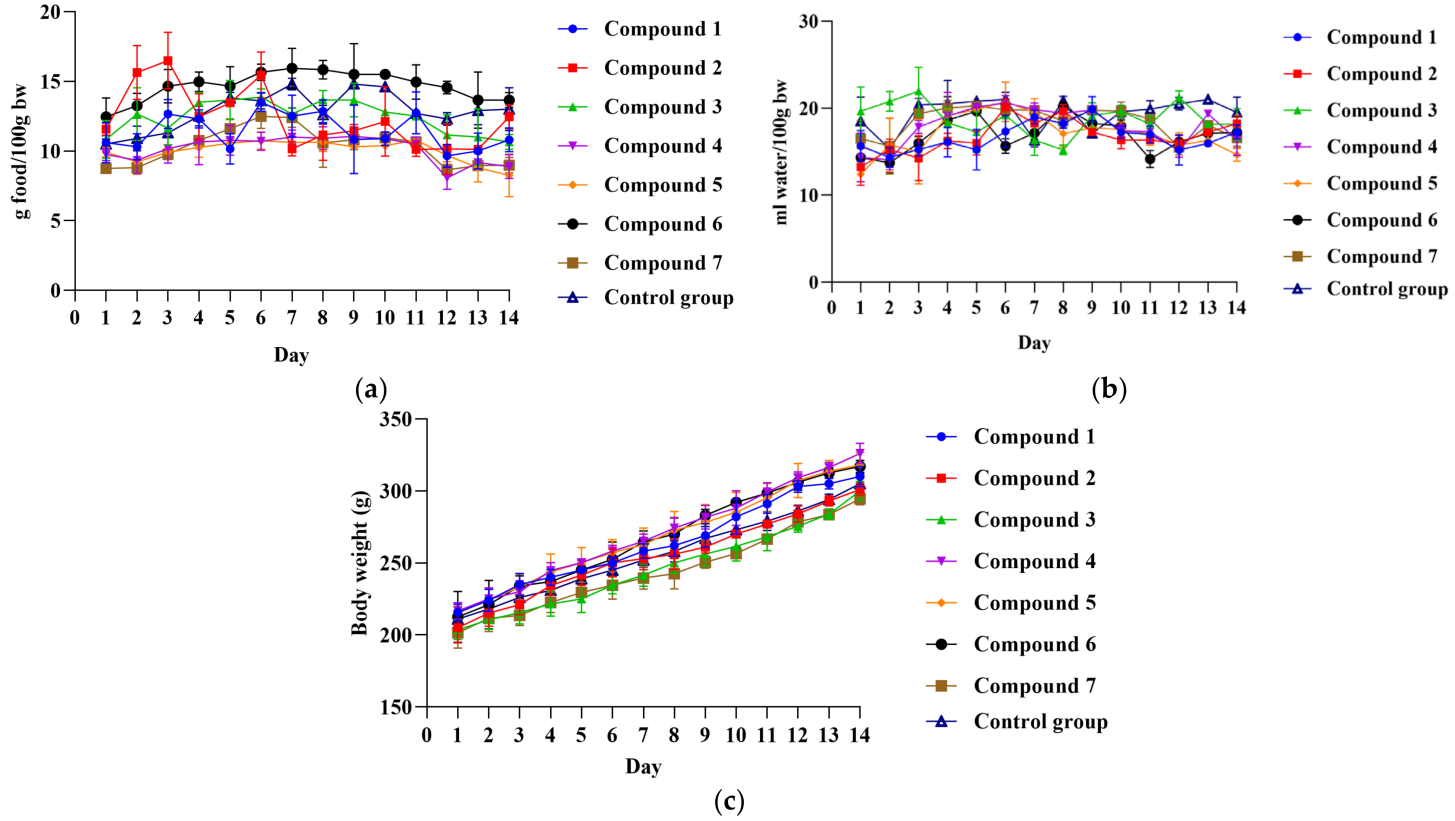
Food intake (**a**), water intake (**b**), and body weight (**c**) in treated and control rats.

No clinical signs of systemic toxicity were observed during 14 days of a careful daily monitoring period in rats that received compounds **1**–**7**. Food and water intake, as well as weight gain, were similar in treated and untreated rats (Figure 1). Importantly, there were no changes in the general appearance and behavior of rats that received compounds **1**–**7**, such as weakness, changes in fur and skin, mucous membrane, urination (color), feces consistency, occurrence of aggressiveness, and changes in locomotor activity. Moreover, there was no statistically significant difference in relative organ weights between treated and control rats (Table 1).

### 2.3. Carrageenan-Induced Paw Edema and Determination of the Anti-Inflammatory Activity

Evaluation of in vivo anti-inflammatory activity of synthesized compounds was carried out using the carrageenan-induced paw edema model of acute inflammation, which is one of the most commonly used models for testing potentially new anti-inflammatory drugs.

**Figure 2:**
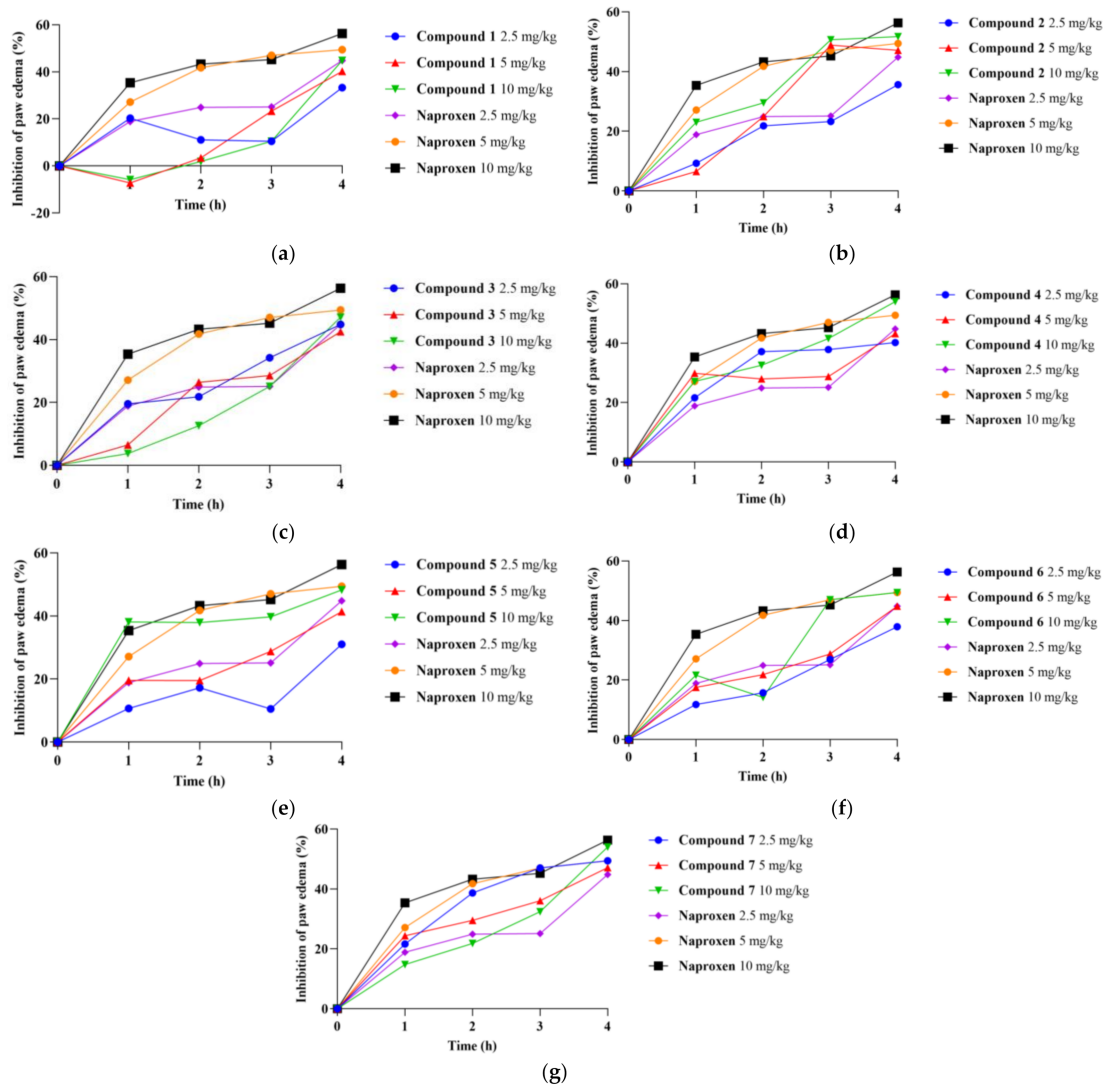
Percentage of paw edema inhibition in rats treated with 2.5 mg/kg, 5 mg/kg, and 10 mg/kg of compound **1** (**a**), compound **2** (**b**), compound **3** (**c**), compound **4** (**d**), compound **5** (**e**), compound **6** (**f**), compound **7** (**g**) and naproxen.

Since compounds **1**–**7** in the highest dose of 10 mg/kg proved to be non-toxic, we aimed to continue in vivo assessment of anti-inflammatory potential by using the carrageenan-induced inflammation model, as it is convenient for testing the effects of different substances to reduce local edema. The carrageenan-induced inflammation in rats was manifested by swelling and quantified by determination of the paw edema elevation. Paw edema increase strongly depended on the time from carrageenan application, i.e., 1, 2, 3, and 4 h and the applied dose of compounds **1**–**7** (2.5, 5 and 10 mg/kg). The results of anti-inflammatory activity for tested compounds are shown in Table 2 and Figure 2.

The first group of compounds, i.e., compounds **1**–**5,** in all doses were capable of decreasing rat paw edema compared to control rats treated with 1% DMSO. The greatest edema inhibition was recorded after three and four hours following carrageenan administration. The most prominent effects were achieved at the fourth hour in compounds **2**–**5** with the percentage inhibition in the range of approximately 47% to 54%. The compound **4** in the highest dose led to a marked drop in paw thickness in the third and fourth hour, as verified by percentage of inhibition of 41.55% and 54.01%, respectively. Moreover, a compound **2** in a dose of 10 mg/kg showed a significant percentage of inhibition of 50.69% and 51.72% after three and four hours following injection of carrageenan. Additionally, compounds **6** and **7** exerted intensive anti-edematous activity, as manifested by percentage inhibition of 49.43% and 54.12% at the fourth hour, respectively.

It was established that acute inflammatory response involves two phases: initial and the later one. The initial phase occurs approximately 1–2 h following carrageenan injection and it is characterized by the histamine and serotonin production. On the other hand, prostaglandins, leukotrienes, and certain cytokines (IL-1β, IL-6, IL-10, and TNF-α) play a significant role in inflammation progress in the later phase [44]. Taking into consideration that compounds **1**–**7** achieved the most prominent effect during the later phase of carrageenan-induced inflammation, we may hypothesize that these compounds affect the release of arachidonate metabolites, probably through alterations of COX and LOX enzyme activity.

It is important to note that anti-inflammatory activity in the current experiment was the greatest after the application of naproxen, while the highest dose of compound **4** in the forth hour exerted a similar effect as this standard drug. In line with that, we can assume that the presence of *m*-substituted aromatic amine attached to thiourea moiety might be associated with prominent anti-inflammatory activity.

A limitation of this in vivo study was the relatively small number of synthesized compounds that limited the ability to perform more detailed structure–activity relationship (SAR) analysis, but provided sufficient data to take initial conclusions about the SAR of these compounds.

### 2.4. Investigation of COX-2 and 5-LOX Enzyme Inhibitory Properties

To evaluate the potential mechanism of anti-inflammatory activity, the inhibitory potential of the synthesized compounds against COX-2 and 5-LOX enzymes was determined using fluorometric COX-2 and 5-LOX inhibition screening kits.

Results of inhibition of COX-2 and 5-LOX enzymes are presented in Table 3 (IC_50_ values and percent of inhibition at 10 µM). For comparison, commercially available selective COX-2 and 5-LOX inhibitors (celecoxib and zileuton, respectively), as well as naproxen, were tested. Results are presented as mean values with standard errors of two measurements.

Neither of the synthesized compounds reached 50% of COX-2 inhibition at concentrations lower than 100 µM, so their COX-2 inhibitory activity is weak. On the other hand, compounds **1**, **2**, **3**, **4**, and **5** could be considered 5-LOX inhibitors and IC_50_ of compound **4** is comparable to zileuton. These findings are in correlation with the results of in vivo study and confirm that one of the possible mechanisms responsible for inflammation reduction achieved by compounds **1**–**5** involves inhibition of 5-LOX, rather than COX-2. Stronger 5-LOX inhibition of derivative **4**, in comparison to other compounds, suggested that the presence of *m*-anisidine in the side chain had significant influence on the achieved inhibitory effect. Moreover, strong 5-LOX inhibition of compound **4**, together with the most intensive inflammation reduction in the rat paw edema model, highlights this compound as a promising anti-inflammatory agent.

### 2.5. Molecular Docking Simulation

In silico investigation of the designed compounds’ binding potential was conducted by molecular docking simulation into the active sites of COX-2 and 5-LOX enzymes. Binding potential was estimated based on the number and type of binding interactions, as well as by free binding energy values.

#### 2.5.1. Molecular Docking of Tested Compounds into COX-2

**Figure 3:**
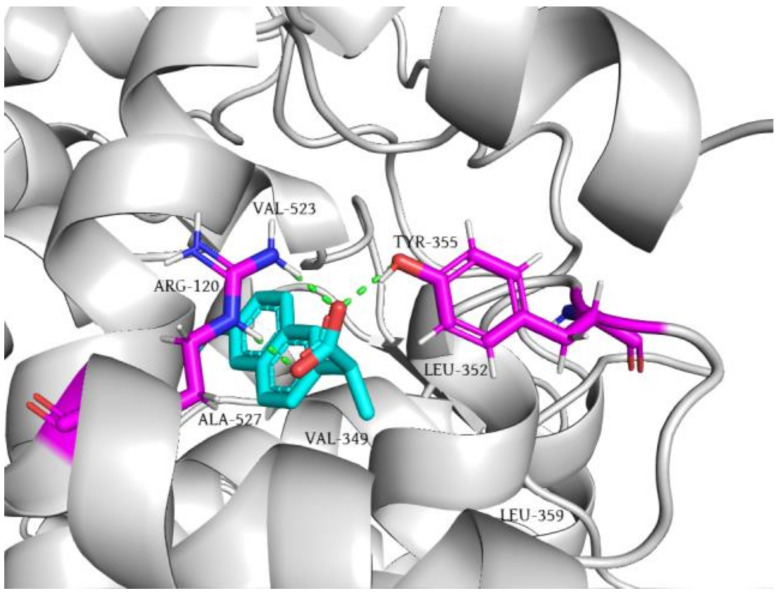
Molecular docking of naproxen into the COX-2 enzyme. The residues involved in the formation of hydrogen bonds (green dashed lines) and hydrophobic interactions are marked.

The interaction of naproxen and COX-2 is characterized by the formation of three hydrogen bonds. The carbonyl and hydroxyl oxygen atoms of the inhibitor’s carboxyl group (hydrogen bond acceptors) interacted with a guanidine group of residue ARG120, while the carbonyl oxygen atom of the inhibitor’s carboxyl group (hydrogen bond acceptor) formed a hydrogen bond with the phenolic group of TYR355 (hydrogen bond donor, Figure 3). In addition, the methoxynaphthalene core of naproxen forms a grid of multiple π-alkyl interactions with residues VAL349, TRP387, LEU352, VAL523, and ALA527, as well as an amide-π stacking interaction with GLY526. Finally, the methyl group of the α-carbon atom establishes alkyl interactions with residues VAL116, VAL349, and LEU531.

**Figure 4:**
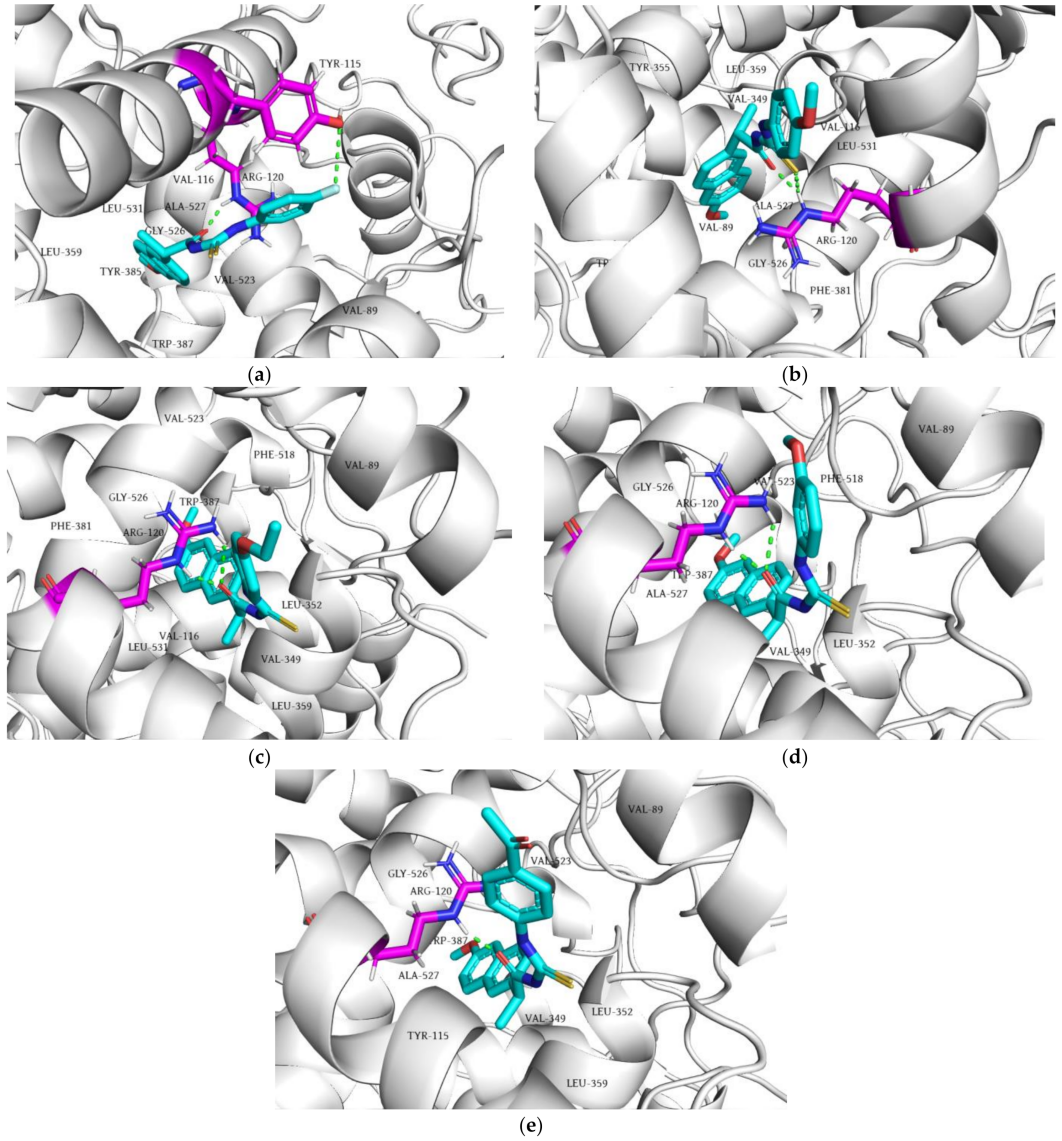
Molecular docking of compound **1** (**a**), compound **2** (**b**), compound **3** (**c**), compound **4** (**d**), and compound **5** (**e**) into the COX-2 enzyme. The residues involved in the formation of hydrogen bonds (green dashed lines) and hydrophobic interactions are marked.

Concerning hydrogen bonds, it can be noticed that the aromatic amine derivatives **1** and **5** form a hydrogen bond with ARG120 via their carbonyl oxygen atoms. On the other hand, the carbonyl and thiocarbonyl groups of derivative **2** and carbonyl group of derivatives **3** and **4** establish a double hydrogen bond with ARG120 (Figure 4).

**Figure 5:**
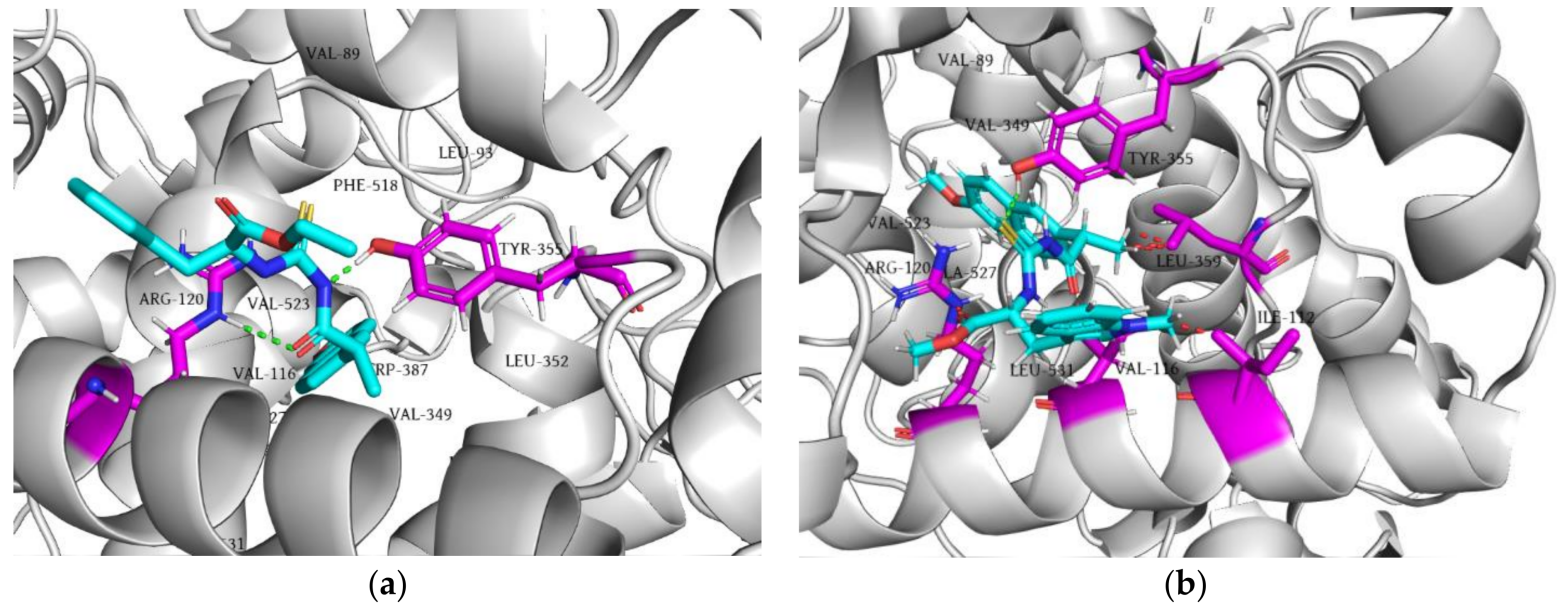
Molecular docking of derivative **6** (**a**) and derivative **7** (**b**) into the COX-2 enzyme. The residues involved in formation of the hydrogen bonds (green dashed lines), hydrophobic interactions, and steric bumps (red dashed lines) are marked.

The phenylalanine ester derivative (**6**) forms hydrogen bonds with ARG120 and TYR355, similar to naproxen, while the *N*-methyl tryptophan ester derivative (**7**) forms a hydrogen bond with TYR355 (Figure 5).

Considering the docking scores of the aromatic amine derivatives, it is noticeable that their values are lower but comparable to naproxen (−13.13 kcal/mol) (Table 4). The comparable docking scores can be explained by the formation of additional binding interactions originating from the aromatic rings of the tested compound’s side chain. Although the methoxynaphthalene moiety of these derivatives was docked into the active site of COX-2 in a similar manner to naproxen, the aromatic rings with their substituents on the opposite side of the molecule contributed significantly to the stabilization of the ligand–protein complex by forming additional interactions with TYR115, VAL89, and VAL116. The lowest docking scores of derivative **4** compared to the other tested compounds can be explained closely by the contacts of the 3-methoxyphenyl residue of the inhibitor with VAL89 (π-σ and alkyl) and VAL116 (π-alkyl).

On the other hand, in comparison to naproxen, the ester derivatives had significantly higher docking scores (Table 4). Despite the two hydrogen bonds formed by compound **6** with ARG120 and TYR355, the benzyl residue in its side chain does not contribute with any binding interaction, while the carbon atoms of the ethyl ester form only alkyl interactions. The low value of the compound’s **7** docking score can be clarified by the occurrence of sterically unfavorable interactions in the enzyme’s active site with ILE112, VAL116, ARG120, and LEU359. The observed steric hindrance can also be explained by the higher voluminosity of this compound compared to naproxen and other tested compounds.

As mentioned before, the carboxyl group of naproxen forms three hydrogen bonds with the side chains of ARG120 and TYR355. These two amino acids, known as constriction site residues, surround the active site of COX-2 and are located at its entrance [45]. The results of mutagenesis studies show that mutations of these constriction site residues completely abolished the COX-2 inhibition induced by naproxen, indicating the importance of these residues and their hydrogen bonds with the inhibitor [46]. Accordingly, the inability of our tested compounds to form three hydrogen bonds with ARG120 and TYR355 might be a reason why these compounds showed weak inhibition of the COX-2 enzyme.

#### 2.5.2. Molecular Docking of the Tested Compounds into 5-LOX

The 5-LOX inhibitors can be divided into three groups according to the nature of their inhibitory action: (a) iron-ligand inhibitors; (b) redox-active inhibitors; and (c) competitive non-redox inhibitors [47]. Zileuton is a 5-LOX inhibitor that achieves its effect by chelating the iron in the active site of the enzyme and/or stabilizing its oxidation state. On the other hand, drugs such as NDGA bind to the active site of the enzyme by competing with arachidonic acid and inhibiting its metabolism [48].

**Figure 6:**
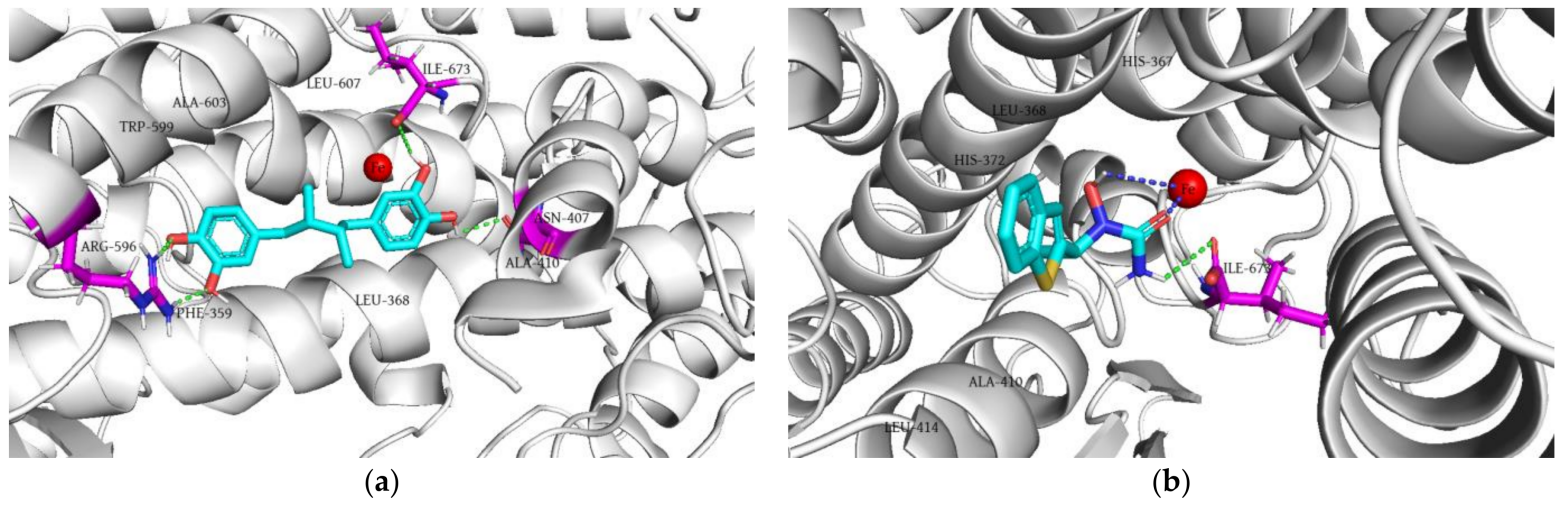
Molecular docking of NDGA (**a**) and zileuton (**b**) into the 5-LOX enzyme. The residues involved in the formation of the hydrogen bonds (green dashed lines) and hydrophobic interactions are marked. Blue dashed lines represent coordination bonds with iron.

To validate the molecular docking study performed on the 5-LOX enzyme, we docked NDGA and zileuton into its active site. The results show that NDGA does not chelate iron (Figure 6a), while zileuton achieves its effect by chelating iron in the active site of 5-LOX (Figure 6b), confirming the validity of the molecular docking study.

**Figure 7:**
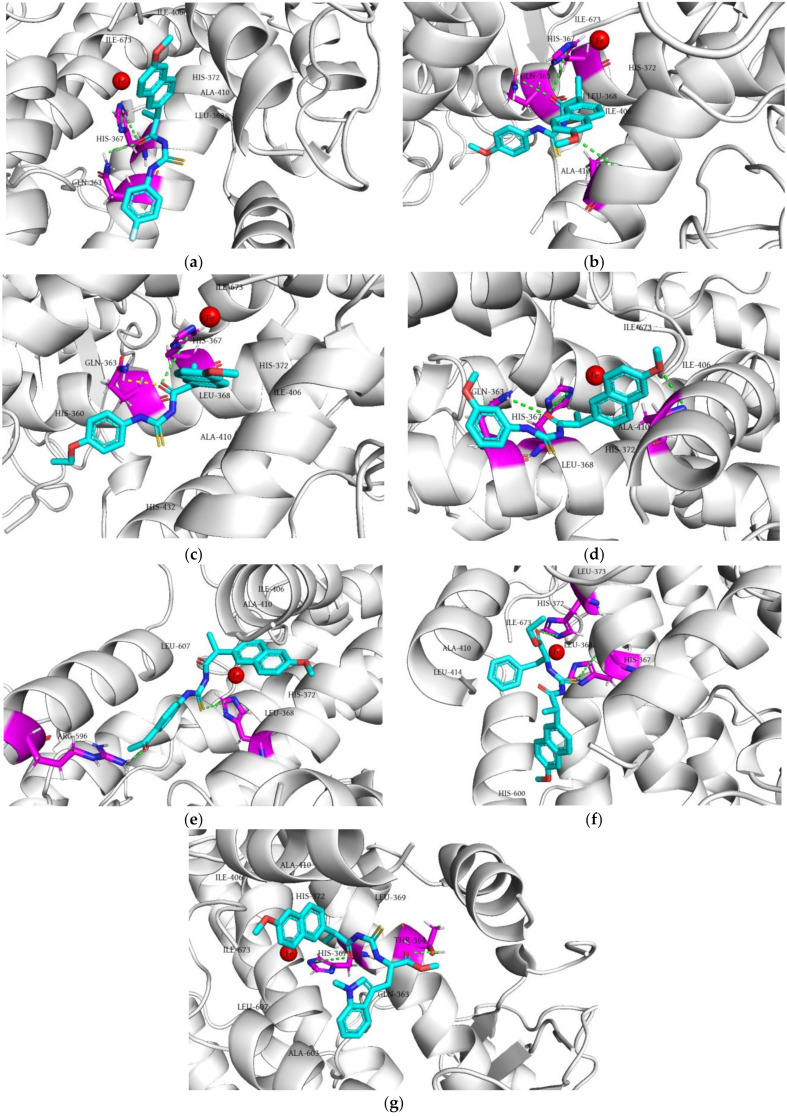
Molecular docking of compound **1** (**a**), compound **2** (**b**), compound **3** (**c**), compound **4** (**d**), compound **5** (**e**), compound **6** (**f**), compound **7** (**g**) into the 5-LOX enzyme. The residues involved in the formation of the hydrogen bonds (green dashed lines) and hydrophobic interactions are marked.

The results of the molecular docking study show that tested compounds (**1**–**7**) are not able to chelate the iron. The naphthalene ring of all tested compounds fits identically in 5-LOX, except for compound **6** (Figure 7). Namely, this part of the molecule forms a π–anion interaction with the ILE673 residue, a π–sigma contact with ALA410, and a π–alkyl interaction with ILE406 in the hydrophobic pocket of the enzyme active site. Based on the presented results, it can be concluded that all compounds showing 5-LOX inhibitory activity in vitro established a hydrogen bond via the carbonyl oxygen of the amide group with the amino acid residue GLN363. Only compound **5** did not show any aforementioned interactions (Figure 7e). The amino acid residue HIS367 forms a hydrogen bond as a hydrogen bond donor with the amide carbonyl oxygen of all compounds, except for derivatives **5** and **6**, where this residue interacts with the sulfur of the thiourea group (Figure 7e,f). The docking scores of all analyzed thiourea derivatives of naproxen were higher than those of NDGA (−10.67 kcal/mol) and zileuton (−8.77 kcal/mol). Among the investigated compounds, compound **4** had the lowest docking score value (−8.39 kcal/mol) (Table 4).

A limitation of conducted molecular docking studies was the usage of semi-flexible docking protocol, which disables consideration of protein-induced fit effects in the presence of ligand.

## 3. Materials and Methods

### 3.1. Chemicals and Instruments

Potassium thiocyanate, phenylalanine ethyl ester hydrochloride, dichloromethane, and *N*,*N*-dimethylformamide were purchased from Acros Organics (Geel, Belgium), whereas *S*-naproxen, oxalyl chloride, *p*-fluoroaniline, *p*-anisidine, *p*-ethoxyaniline, *m*-anisidine, *p*-aminoacetophenone, acetone, and carrageenan were purchased from Sigma-Aldrich (Steinheim, Germany). Chloroform and methanol were purchased from JT Baker (Loughborough, UK), whereas silica gel for preparative thin-layer chromatography was obtained from Merck (Darmstadt, Germany). 

The synthesized compounds were structurally characterized by determining melting points and by spectroscopic methods (IR, NMR, HRMS, and MS/MS). Melting points were determined using the Boetius PHMK 05 apparatus (Radebeul, Germany). The ^1^H NMR and ^13^C NMR spectra were recorded on an NMR Bruker Avance III spectrometer (Bruker Biospin GmbH, Rheinstetten, Germany), operated at 400 MHz (^1^H NMR) and 100 MHz (^13^C NMR). IR spectra were recorded using the ATR-FTIR spectrometer Nicolet iS10 (Thermo Scientific, Madison, WI, USA). Exact masses were determined using the LTQ Orbitrap XL Mass Spectrometer (Thermo Fisher Scientific, Bremen, Germany). MS/MS analyses were performed using the TSQ Quantum Access MAX triple quadrupole mass spectrometer (Thermo Fisher Scientific, San Jose, CA, USA), equipped with a heated electrospray ionization source (HESI).

### 3.2. Synthetic Procedures

As shown in Scheme 1, the series of naproxen thiourea derivatives (**1**–**7**) was synthetized by a three-step procedure. In the first step, *S*-naproxen (250 mg, 1.0857 mmol,) was dissolved in dichloromethane (CH_2_Cl_2_, 5 mL), and oxalyl-chloride (186 μL, 2.1714 mmol) was added dropwise with a catalytic amount of DMF (three drops) at room temperature. The mixture was stirred at the same temperature for three hours. The reaction mixture was evaporated to dryness under reduced pressure to give corresponding acyl chloride. In the second step, the solution of the acyl chloride (125 mg, 0.5 mmol) in anhydrous acetone (3.5 mL) was added dropwise to a suspension of potassium-thiocyanate (48.59 mg, 0.5 mmol) in acetone (3.5 mL) and the reaction mixture was refluxed for one hour. Finally, aromatic amines or aromatic amino acid esters were added into the mixture (third step). Compounds **1**–**5** were synthesized by adding a solution of 0.5 mmol of aromatic amine (*p*-fluoroaniline for compound **1**, *p*-anisidine for compound **2**, *p*-ethoxyaniline for compound **3**, *m*-anisidine for compound **4**, and *p*-aminoacetophenone for compound **5**) in anhydrous acetone (3.5 mL) into the mixture. On the other hand, compounds **6** and **7** were synthesized by adding a suspension of 0.5 mmol of amino acid ester hydrochloride (phenylalanine ethyl ester for compound **6** and *N*-methyl tryptophan methyl ester for compound **7**) and an equimolar amount of triethylamine (TEA, 0.5 mmol) in acetone (3.5 mL) into the mixture. The final reaction mixtures were refluxed for three hours and then evaporated to dryness at room temperature.

The reaction mixtures were then dissolved in chloroform and purified by preparative thin-layer chromatography (TLC). The mobile phases used for preparative thin-layer chromatography were chloroform (aromatic amines) and chloroform/methanol 99:1, *v*/*v* (aromatic amino acid esters).

*(S)-N-((4-fluorophenyl)carbamothioyl)-2-(6-methoxynaphthalen-2-yl)propanamide* (**1**)

Yield: 28%. Yellow crystalline solid. Melting point: 57–59 °C. IR (ATR) ν_max_ (cm^−1^): 1145.06, 1686.94, 2934.27, and 3168.25. **^1^H NMR (400 MHz, DMSO-d_6_) δ ppm** 1.51 (3H, d, J = 6.8 Hz, C**H_3_**_Nap_), 3.87 (3H, s, OC**H_3_**_Nap_), 4.22 (1H, q, J = 6.8 Hz, C**H**_Nap_), 7.16–7.85 (10H, m, Ar**H**), 11.72 (1H, s, N**H**CS), and 12.31 (1H, s, CSN**H**) (Figure S1a). **^13^C NMR (100 MHz, DMSO-d_6_) δ ppm** 17.45 (**C**H_3Nap_), 44.35 (**C**H_Nap_), 54.59 (O**C**H_3Nap_), 105.12, 114.71 (CF**C**HCHCH-, J = 22.6 Hz), 118.26, 125.36, 125.66, 126.31 (CFCH**C**HCH-, J = 8.4 Hz), 126.36, 126.45, 127.74, 128.65, 132.88, 133.51 (CFCHCH**C**H-, J = 2.9 Hz), 134.66, 156.69, 159.40 (**C**FCHCHCH-, J = 242.3), 175.46 (**C**ONH), and 178.97 (NH**C**S) (Figure S1b). *m/z* = 383.1, [M+H]^+^, 229.97, 185.02, 170.96, 169.05, 154.00, and 112.14. MS [M+Na]^+^ calculated for C_21_H_19_FN_2_O_2_S = 405.10435; observed = 405.10432.

*(S)-2-(6-methoxynaphthalen-2-yl)-N-((4-methoxyphenyl)carbamothioyl)propanamide* (**2**)

Yield: 33%. Yellow crystalline solid. Melting point: 112–114 °C. IR (ATR) ν_max_ (cm^−1^): 1146.07, 1686.55, 2932.67, and 3184.85. **^1^H NMR (400 MHz, DMSO) δ ppm** 1.49 (3H, d, J = 6.4 Hz, C**H_3_**_Nap_), 3.74 (3H, s, OC**H_3_**), 3.86 (3H, s, OC**H_3_**_Nap_), 4.21 (1H, q, J = 6.4 Hz, C**H**_Nap_), 6.90–7.82 (10H, m, Ar**H**), 11.61 (1H, s, N**H**CS), and 12.26 (1H, s, CSN**H**) (Figure S2a). **^13^C NMR (100 MHz, DMSO-d_6_) δ ppm** 18.51 (**C**H_3Nap_), 45.40 (**C**H_Nap_), 55.65 (O**C**H_3Nap_), 55.73 (O**C**H_3_), 106.19, 114.22, 119.31, 126.37, 126.40, 126.71, 127.51, 128.81, 129.71, 131.03, 133.94, 135.79, 157.75, 157.89 (aromatic carbons), 176.56 (**C**ONH), and 179.52 (NH**C**S) (Figure S2b). *m/z* = 394.9 [M+H]^+^, 230.03, 184.99, 183.01, 169.78, 153.11, and 124.10. MS [M+Na]^+^ calculated for C_22_H_22_N_2_O_3_S = 417.12433; observed = 417.12418.

*(S)-N-((4-ethoxyphenyl)carbamothioyl)-2-(6-methoxynaphthalen-2-yl)propanamide* (**3**)

Yield: 31%. Yellow crystalline solid. Melting point: 115–116 °C. IR (ATR) ν_max_ (cm^−1^): 1142.79, 1677.97, 2935.29, and 3255.39. **^1^H NMR (400 MHz, DMSO) δ ppm** 1.31 (3H, t, J = 7.2 Hz, OCH_2_C**H_3_**), 1.50 (3H, d, J = 6.8 Hz, C**H_3_**_Nap_), 3.87 (3H, s, OC**H_3_**_Nap_), 4.01 (2H, q, J = 6.8 Hz, OC**H_2_**CH_3_), 4.22 (1H, q, J = 6.8 Hz, C**H**_Nap_), 6.90–7.85 (10H, m, Ar**H**), 11.63 (1H, s, N**H**CS), and 12.27 (1H, s, CSN**H**) (Figure S3a). **^13^C NMR (100 MHz, DMSO-d_6_) δ ppm** 14.02 (OCH_2_**C**H_3_), 17.46 (**C**H_3Nap_), 44.34 (**C**H_Nap_), 54.59 (O**C**H_3Nap_), 62.61 (O**C**H_2_CH_3_), 105.13, 113.60, 118.24, 125.25, 125.34, 125.66, 126.44, 127.75, 128.65, 129.84, 132.88, 134.73, 156.11, 156.69 (aromatic carbons), 175.49 (**C**ONH), and 178.40 (NH**C**S) (Figure S3b). *m/z* = 409.0 [M+H]^+^, 229.92, 196.92, 184.99, 179.93, 170.01, and 153.04. MS [M+Na]^+^ calculated for C_23_H_24_N_2_O_3_S = 431.13998; observed = 431.14020.

*(S)-2-(6-methoxynaphthalen-2-yl)-N-((3-methoxyphenyl)carbamothioyl)propanamide* (**4**)

Yield: 43%. Green crystalline solid. Melting point: 144–145 °C. IR (ATR) ν_max_ (cm^−1^): 1155.54, 1683.27, 3014.24, and 3224.80. **^1^H NMR (400 MHz, DMSO) δ ppm** 1.50 (3H, d, J = 6.8 Hz, C**H_3_**_Nap_), 3.72 (3H, s, OC**H_3_**), 3.86 (3H, s, OC**H_3_**_Nap_), 4.21 (1H, q, J = 7.2 Hz, C**H**_Nap_), 6.79–7.84 (10H, m, Ar**H**), 11.68 (1H, s, N**H**CS), and 12.46 (1H, s, CSN**H**) (Figure S4a). **^13^C NMR (100 MHz, DMSO-d_6_) δ ppm** 18.50 (**C**H_3Nap_), 45.44 (**C**H_Nap_), 55.65 (O**C**H_3Nap_), 55.36 (O**C**H_3_), 106.20, 110.07, 112.45, 116.65, 119.31, 126.42, 126.72, 127.52, 128.81, 129.71, 129.89, 133.95, 135.72, 139.23, 157.76, 159.76 (aromatic carbons), 176.67 (**C**ONH), and 179.19 (NH**C**S) (Figure S4b). *m/z* = 395.2 [M+H]^+^, 230.10, 185.08, 183.03, 170.07, 154.11, and 153.11. MS [M+H]^+^ calculated for C_22_H_22_N_2_O_3_S = 395.14239; observed = 395.14197.

*(S)-N-((4-acetylphenyl)carbamothioyl)-2-(6-methoxynaphthalen-2-yl)propanamide* (**5**)

Yield: 38%. Yellow crystalline solid. Melting point: 132–134 °C. IR (ATR) ν_max_ (cm^−1^): 1145.15, 1685.96, 2988.46, and 3180.83. **^1^H NMR (400 MHz, DMSO) δ ppm** 1.52 (3H, d, J = 6.8 Hz, C**H_3_**_Nap_), 2.56 (3H, s, C(=O)C**H_3_**), 3.87 (3H, s, OC**H_3_**_Nap_), 4.23 (1H, q, J = 7.2 Hz, C**H**_Nap_), 7.16–7.97 (10H, m, Ar**H**), 11.80 (1H, s, N**H**CS), 12.67 (1H, s, CSN**H**) (Figure S5a). **^13^C NMR (100 MHz, DMSO-d_6_) δ ppm** 17.47 (**C**H_3Nap_), 26.08 (C(=O)**C**H_3_), 44.43 (**C**H_Nap_), 54.59 (O**C**H_3Nap_), 105.14, 118.26, 122.87, 125.38, 125.66, 126.47, 127.75, 128.25, 128.65, 132.89, 133.61, 134.58, 141.24, 156.71 (aromatic carbons), 175.60 (**C**ONH), 178.26 (NH**C**S), and 196.22 (**C**(=O)CH_3_) (Figure S5b). *m/z* = 407.2 [M+H]^+^, 230.07, 195.05, 185.11, 170.09, 152.36, and 136.14. MS [M+Na]^+^ calculated for C_23_H_22_N_2_O_3_S = 429.12433; observed = 429.12489.

*Ethyl(((S)-2-(6-methoxynaphthalen-2-yl)propanoyl)carbamothioyl)-L-phenylalaninate* (**6**)

Yield: 30%. Yellow crystalline solid. Melting point: 59–61 °C. IR (ATR) ν_max_ (cm^−1^): 1155.75, 1688.89, 1737.73, 2934.28, and 3197.26. **^1^H NMR (400 MHz, DMSO-d_6_) δ ppm** 1.16 (3H, t, J = 7.2 Hz, R-C(=O)OCH_2_C**H_3_**), 1.43 (3H, d, J = 6.8 Hz, C**H_3_**_Nap_), 3.15 (2H, dd, J = 5.6 Hz, J = 10 Hz, C**H_2_**_-Phe_), 3.87 (3H, s, OC**H_3_**_Nap_), 4.10 (1H, q, J = 7.2 Hz, C**H**_Nap_), 4.13 (2H, q, J = 3.6 Hz, R-C(=O)OC**H_2_**CH_3_), 5.04 (1H, q, J = 6.4 Hz, C**H**_-Phe_) 7.07–7.83 (11H, m, Ar**H**), 10.98 (1H, s, N**H**CS), 11.57 (1H, s, CSN**H**) (Figure S6a). **^13^C NMR (100 MHz, DMSO-d_6_) δ ppm** 14.39 (R-C(=O)OCH_2_**C**H_3_), 18.60 (**C**H_3Nap_), 36.71 (**C**H_2-Phe_), 45.41 (**C**H_Nap_), 55.68 (O**C**H_3Nap_), 58.94 (**C**H_-Phe_), 61.59 (R-C(=O)O**C**H_2_CH_3_), 106.27, 119.3, 126.27, 126.49, 127.36, 127.51, 128.75, 128.83, 129.59, 129.65, 133.92, 135.90, 136.19, 157.78 (aromatic carbons), 170.30 (R-**C**(=O)OCH_2_CH_3_), 176.50 (**C**ONH), and 180.77 (NH**C**S) (Figure S6b). *m/z* = 465.00 [M+H]^+^, 418.86, 390.73, 184.95, 178.93, 169.96, and 120.08. MS [M+Na]^+^ calculated for C_26_H_28_N_2_O_4_S = 487.16620; observed = 487.16578.

*Methyl-N^α^-(((S)-2-(6-methoxynaphthalen-2-yl)propanoyl)carbamothioyl)-1-methyltryptophanate* (**7**)

Yield: 25%. Light-yellow crystalline solid. Melting point: 88–90 °C. IR (ATR) ν_max_ (cm^−1^): 1174.36, 1693.37, 1740.80, 2932.10, and 3188.46. **^1^H NMR (400 MHz, DMSO) δ ppm** 1.40 (3H, d, J = 7.2 Hz, C**H_3_**_Nap_), 3.20 (2H, dd, J = 4.0 Hz, J = 12 Hz, C**H_2_**_-Trp_), 3.62 (3H, s, NC**H_3_**-Trp), 3.63 (3H, s, R-C(=O)OC**H_3_**), 3.87 (3H, s, OC**H_3_**_Nap_), 4.10 (1H, q, J = 7.2 Hz, C**H**_Nap_), 5.06 (1H, q, J = 5.6 Hz, C**H**_-Trp_), 6.67–7.81 (11H, m, Ar**H**), 10.99 (1H, s, N**H**CS), and 11.59 (1H, s, CSN**H**) (Figure S7a). **^13^C NMR (100 MHz, DMSO-d_6_) δ ppm** 18.54 (**C**H_3Nap_), 26.72 (**C**H_2-Trp_), 32.67 (N**C**H_3_-Trp), 45.30 (**C**H_Nap_), 52.76 (R-C(=O)O**C**H_3_), 55.68 (O**C**H_3Nap_), 58.90 (**C**H_-Trp_), 106.21, 107.78, 110.09, 118.47, 119.07, 119.27, 121.56, 126.33, 126.56, 127.49, 127.84, 128.71, 128.82, 129.71, 133.94, 135.81, 136.88, 157.75 (aromatic carbons), 171.13 (R-**C**(=O)OCH_3_), 176.44 (**C**ONH), and 180.61 (NH**C**S) (Figure S7b). *m/z* = 504.3 [M+H]^+^, 470.36, 275.05, 216.07, 185.09, 174.11, and 144.14. MS [M+Na]^+^ calculated for C_28_H_29_N_3_O_4_S = 526.17710; observed = 526.17742.

### 3.3. Evaluation of Toxicity Profile and Anti-Inflammatory Activity in Wistar Albino Rats

The acute oral toxicity study and in vivo examination of anti-inflammatory activity of compounds **1**–**7** were conducted at the Faculty of Medical Sciences, University of Kragujevac, Serbia. Rats were obtained from the Military Medical Academy, Belgrade, Serbia and housed at a temperature of 22 ± 2 °C, with 12 h of automatic illumination daily. Animals consumed commercial rat food (20% protein rat food; Veterinary Institute Subotica, Serbia) and water ad libitum. The study protocol was performed in accordance with the regulations of the Faculty’s Ethics Committee for the welfare of laboratory animals (protocol code 01-10742, approval date: 14 October 2021) and principles of the Good Laboratory Practice and European Council Directive (86/609/EEC).

#### 3.3.1. Acute Oral Toxicity Evaluation

Healthy male rats (*n* = 24, three rats per group, body weight 180–200 g) were used for conducting an acute oral toxicity study. Animals were divided into the following groups: a control group that included rats that received a single dose of 1% DMSO and experimental groups that involved rats that received compounds **1**–**7** in the single dose of 10 mg/kg. 

After oral administration of the aforementioned agents, rats were kept in single cages and monitored during 14 days. Food and water intake, as well as body weight, were measured daily. All the behavioral changes and any signs of toxicity were monitored daily during 2-week protocol (fur and skin, eyes, salivation, respiration, urination (color), faeces consistency, somatomotor activity, and other alterations in behavior pattern). After accomplishing 14 days, animals were sacrificed and organ weight (liver, kidney, stomach, and heart) was recorded [49,50]. Relative organ weight was calculated as follows:

(absolute organ weight × 100%)/body weight of rat on the day of sacrifice.(1)

#### 3.3.2. Evaluation of Anti-Inflammatory Activity in Wistar Albino Rats

In vivo assessment of anti-inflammatory activity of compounds **1**–**7** was conducted in the carrageenan-induced rat paw edema model. Inflammation in all rats was induced by an intraplantar injection of 1 mL of 0.5% carrageenan saline in the left hind paw. Two hundred male Wistar albino rats (180–210 g) were divided into seven experimental (*n* = 168 rats, 8 rats per subgroup) and two control groups (*n* = 32 rats, 8 rats per subgroup). 

Experimental groups involved rats that received compounds **1**–**7**, and they were divided into three subgroups: rats received compounds **1**–**7** per os in three doses (2.5 mg/kg, 5 mg/kg, and 10 mg/kg) dissolved in 1% DMSO for 60 min before inducing inflammation.

Control groups involved: 1% DMSO group—rats were treated per os with 1% DMSO (the volume of applied 1% DMSO was the same as the volume of compounds **1**–**7**) sixty minutes before inducing inflammation and naproxen groups—rats received naproxen in three doses (2.5 mg/kg, 5 mg/kg, and 10 mg/kg) per os dissolved in 1% DMSO sixty minutes before inducing inflammation. Doses of tested compounds and naproxen were selected based on previously published research [35,51].

In order to quantify the anti-inflammatory effect, the thickness of the left paw tissue of each rat was measured at the following time intervals: immediately before inducing inflammation (moment 0) and 1, 2, 3, and 4 h (moments 1, 2, 3, and 4) after inflammation. Tissue thickness was measured in the middle of the rat paw using a digital vernier caliper (Aerospace, Beijing, China). The percentage of inhibition of the paw edema was calculated according to the formula:

% Inhibition = 100 × [1 − (Yt/Yc)](2)

where Yt = average increase in paw thickness in the treated group of rats between two measurement moments, and Yc = average increase in paw thickness in the untreated group of rats between two measurement moments [52].

### 3.4. Investigation of COX-2 and 5-LOX Inhibitory Activity

COX-2 and 5-LOX inhibitory activities were tested using fluorometric COX-2 and 5-LOX inhibitor screening kits (Abcam, UK). These assays are based on the fluorometric detection of Prostaglandin G2 (a product generated by the COX enzyme) or on the fluorometric detection of intermediate generated by the 5-LOX enzyme. The experiments were conducted according to the manufacturers’ instructions [53,54].

For the determination of COX-2 inhibitory activity, tested compounds were dissolved in DMSO (5 mM stock solutions) and then diluted with the same solvent to obtain test solutions. Test solutions were further diluted 5X with COX assay buffer (2 µL of test solution and 8 µL COX assay buffer). Inhibitor control (IC) was prepared by adding 2 µL celecoxib (selective COX-2 inhibitor) and 8 µL COX assay buffer into corresponding wells. Solvent control (SC) was prepared by adding 2 µL DMSO and 8 µL COX assay buffer, while sample (S) and enzyme control (EC) were prepared by adding 10 µL of diluted test solution and COX assay buffer, respectively, into assigned wells. For the determination of 5-LOX inhibitory activity, tested compounds were also dissolved in DMSO to prepare stock solutions with the same concentration (5mM), which were then diluted with the same solvent to obtain test solutions. Subsequently, test solutions were added to corresponding wells (2 µL). EC, SC, and IC were prepared by adding 2 µL of assay buffer, DMSO, and zileuton into corresponding wells, respectively. 

Fluorescence of the samples were kinetically measured at 25 °C (COX-2: Ex/Em = 535/587 nm, during 10 min; 5-LOX: Ex/Em = 500/536 nm during 20 min) using the Synergy LX multi-mode microplate reader (BioTek, Winooski, VT, USA).

### 3.5. Molecular Docking Studies

For the binding affinity assessment of compounds **1**–**7** towards COX-2 and 5-LOX enzymes, the following crystal structures were obtained from the Protein Data Bank [55]: 3NT1 (naproxen bound to COX-2) [46] and 6N2W (NDGA bound to 5-LOX) [56]. MAKE Receptor 3.2.0.2 software was used for the preparation of target enzymes [57]. The molecular docking analyses were carried out in the boxes generated around co-crystallized ligands, the dimensions of which were 33.67 Å × 27.33 Å × 23.33 Å (COX-2) and 19.67 Å × 14.67 Å × 22.67 Å (5-LOX). The outer contour sizes were 617 Å^3^ (COX-2) and 908 Å^3^ (5-LOX). The site shape setup was defined as balanced without defined constraints.

The multiconformer compound data sets were prepared in OMEGA 2.5.1.4 [58,59]. The OEDocking 3.2.0.2 software [60,61,62], with the fast rigid exhaustive docking (FRED) tool, was utilized for the binding analysis of ligand conformations into the active sites of target enzymes. The docked conformers were scored using the Chemgauss4 [63] scoring function, while further optimization was performed using the OEChemscore [64] scoring function. The Chemgauss4 scoring function was used for scoring and consensus pose selection, whereas other settings were set as default.

Native conformations of naproxen and NDGA were re-docked into the active sites of COX-2 and 5-LOX to conduct the validation of docking protocol. Conformers of co-crystallized ligands obtained by molecular docking were superimposed with their crystallographic binding poses, and root-mean-square deviation (RMSD) values were calculated. The calculated RMSD values were less than 2.0 Å, which confirmed the validity of the conducted molecular docking experiments [65].

## 4. Conclusions

In this paper, we obtained a series of seven thiourea derivatives of naproxen with selected aromatic amines and aromatic amino acid esters that were not previously synthesized and characterized. The results of in vivo study indicate that compounds **2**–**5** showed the most pronounced anti-inflammatory effect four hours following injection of carrageenan. The compounds **2** and **4** in the highest dose demonstrated a significant percentage of inhibition of 50.69% and 41.55% after three hours, and 51.72% and 54.01% after four hours of carrageenan application, respectively. On the other hand, amino acid ester derivatives **6** and **7** in the same dose led to a significant reduction in paw thickness in the fourth hour, with a percentage of inhibition of 49.43% and 54.12%, respectively. The results of in vitro assays of COX-2 inhibition reveal that none of the tested compounds achieved 50% of COX -2 inhibition at concentrations lower than 100 µM. In line with that, the inability of tested compounds to form three hydrogen bonds with ARG120 and TYR355 might be a reason why these compounds showed weak COX-2 inhibition. On the contrary, compounds **1**–**5** could be considered 5-LOX inhibitors, whereby IC_50_ of compound **4** (0.30 μM) was comparable to that of the commercial 5-LOX inhibitor zileuton (0.36 μM). The results of in vivo anti-inflammatory activity reveal that aromatic amine derivatives generally exhibited a stronger anti-inflammatory effect in comparison to aromatic amino acid ester derivatives. Data obtained by in vitro enzyme inhibition assays and the molecular docking study confirm these findings and highlight *m*-substituted compound **4** as the derivative with the most potent anti-inflammatory effect. Given that the presented compounds were not previously synthesized, results presented in this paper (regarding their anti-inflammatory activity, inhibitory activity against COX-2 and 5-LOX, as well as their interaction with these enzymes) will facilitate the design of new potent anti-inflammatory compounds. However, a relatively small set of synthesized compounds provided sufficient data to take only initial conclusions about the SAR of these compounds.

## Figures and Tables

**Table 1:** Relative organ weights in treated and control rats.

Compound	1	2	3	4	5	6	7	Control Group
Organ								
Kidney	0.31 ± 0.03	0.34 ± 0.04	0.32 ± 0.06	0.33 ± 0.07	0.30 ± 0.03	0.35 ± 0.05	0.29 ± 0.03	0.33 ± 0.01
Heart	0.32 ± 0.02	0.33 ± 0.04	0.31 ± 0.03	0.30 ± 0.03	0.31 ± 0.03	0.34 ± 0.02	0.33 ± 0.03	0.31 ± 0.01
Liver	2.73 ± 0.24	3.07 ± 0.38	2.85 ± 0.17	2.45 ± 0.15	2.97 ± 0.33	3.01 ± 0.29	2.64 ± 0.46	2.91 ± 0.10
Stomach	0.54 ± 0.05	0.57 ± 0.04	0.50 ± 0.05	0.50 ± 0.05	0.53 ± 0.04	0.55 ± 0.03	0.50 ± 0.04	0.53 ± 0.04

**Table 2:** Anti-inflammatory activity of tested compounds in the carrageenan-induced rat paw edema model. The results are presented as the mean value ± standard deviation (SD).

Rat Paw Thickness (mm) (% of Inhibition)					
Experimental Groups	0 h	1 h	2 h	3 h	4 h
**Compound 1** 2.5 mg/kg	4.57 ± 0.31	6.50 ± 0.40 (20.275%)	6.50 ± 0.40 (11.111%)	6.20 ± 0.30 (10.502%)	5.53 ± 0.29 (33.333%)
**Compound 1** 5.0 mg/kg	4.40 ± 0.36	7.00 ± 0.10 (−7.216%)	6.50 ± 0.17 (3.448%)	5.8 ± 0.20 (23.288%)	5.27 ± 0.29 (40.230%)
**Compound 1** 10.0 mg/kg	4.47 ± 0.51	7.03 ± 0.12 (−5.842%)	6.60 ± 0.10 (1.916%)	6.10 ± 0.17 (10.502%)	5.27 ± 0.50 (44.800%) *
**Compound 2** 2.5 mg/kg	4.37 ± 0.15	6.57 ± 1.16 (9.278%)	6.07 ± 0.72 (21.839%)	5.77 ± 0.42 (23.288%)	5.30 ± 0.26 (35.632%)
**Compound 2** 5.0 mg/kg	4.77 ± 0.21	7.03 ± 0.47 (6.529%)	6.40 ± 0.53 (24.904%)	5.70 ± 0.20 (48.858%) *	5.53 ± 0.29 (47.126%) *
**Compound 2** 10.0 mg/kg	4.63 ± 0.06	6.5 ± 0.40 (23.024%)	6.17 ± 0.67 (29.502%)	5.53 ± 0.15 (50.685%) *	5.33 ± 0.25 (51.724%) *
**Compound 3** 2.5 mg/kg	4.30 ± 0.28	6.25 ± 0.21 (19.588%)	6.0 ± 0.57 (21.839%)	5.50 ± 0.42 (34.247%)	5.10 ± 0.14 (44.737%)
Compound 3 5.0 mg/kg	4.13 ± 0.25	6.40 ± 0.79 (6.529%)	5.73 ± 0.38 (26.437%) *	5.44 ± 0.28 (28.584%)	4.97 ± 0.42 (42.529%) *
**Compound 3** 10.0 mg/kg	3.90 ± 0.20	6.23 ± 0.25 (3.780%)	5.80 ± 0.26 (12.644%)	5.27 ± 0.15 (25.114%)	4.67 ± 0.38 (47.217%) *
**Compound 4** 2.5 mg/kg	4.03 ± 0.06	5.93 ± 0.25 (21.649%)	5.40 ± 0.10 (37.165%) *	5.17 ± 0.15 (37.900%)	4.90 ± 0.10 (40.523%) *
**Compound 4** 5.0 mg/kg	4.40 ± 0.62	6.10 ± 0.44 (29.897%) *	5.97 ± 0.25 (27.969%)	5.70 ± 0.46 (28.767%)	5.22 ± 0.50 (43.218%) *
**Compound 4** 10.0 mg/kg	4.23 ± 0.06	6.00 ± 0.10 (27.148%) *	5.70 ± 0.20 (32.567%) *	5.30 ± 0.17 (41.553%) *	4.90 ± 0.20 (54.013%) *
**Compound 5** 2.5 mg/kg	3.57 ± 0.12	5.73 ± 0.12 (10.653%)	5.37 ± 0.25 (17.241%)	5.20 ± 0.20 (10.502%)	4.57 ± 0.15 (31.034%)
**Compound 5** 5.0 mg/kg	3.10 ± 0.00	5.05 ± 0.07 (19.588%)	4.85 ± 0.07 (19.540%)	4.40 ± 0.14 (28.767%)	3.95 ± 0.07 (41.379%)
**Compound 5** 10.0 mg/kg	4.00 ± 0.28	5.5 ± 0.71 (38.144%) *	5.35 ± 0.64 (37.931%) *	5.90 ± 0.71 (39.726%)	4.75 ± 0.49 (48.276%)
**Compound 6** 2.5 mg/kg	4.03 ± 0.06	6.17 ± 0.31 (11.753%)	5.87 ± 0.23 (15.709%)	5.37 ± 0.55 (26.941%)	4.93 ± 0.21 (37.931%)
**Compound 6** 5.0 mg/kg	4.47 ± 0.35	6.47 ± 0.57 (17.526%)	6.17 ± 0.06 (21.839%)	5.77 ± 0.06 (28.767%)	5.27 ± 0.21 (44.724%)
**Compound 6** 10.0 mg/kg	4.30 ± 0.62	6.20 ± 0.20 (21.649%)	6.17 ± 0.23 (14.176%)	5.27 ± 0.72 (47.103%)	5.03 ± 0.76 (49.425%) *
**Compound 7** 2.5 mg/kg	5.13 ± 0.29	7.03 ± 0.15 (21.649%)	6.47 ± 0.21 (38.697%) *	6.10 ± 0.20 (47.032%) *	5.87 ± 0.21 (49.425%) *
**Compound 7** 5.0 mg/kg	5.27 ± 0.40	7.10 ± 0.17 (24.399%)	6.80 ± 0.20 (29.502%) *	6.43 ± 0.32 (36.073%)	6.03 ± 0.61 (47.126%)
**Compound 7** 10.0 mg/kg	4.80 ± 0.53	6.87 ± 0.25 (14.777%)	6.50 ± 0.36 (21.839%)	6.03 ± 0.21 (32.420%)	5.47 ± 0.38 (54.123%) *
**1% DMSO**	4.40 ± 0.08	6.83 ± 0.30	6.58 ± 0.30	6.23 ± 0.46	5.85 ± 0.31
**Naproxen** 2.5 mg/kg	4.23 ± 0.40	6.20 ± 0.10 (18.900%)	5.87 ± 0.12 (24.904%)	5.60 ± 0.17 (25.114%)	5.03 ± 0.25 (44.828%)
**Naproxen** 5.0 mg/kg	4.10 ± 0.10	5.87 ± 0.23 (27.148%)	5.37 ± 0.31 (41.762%)	5.07 ± 0.35 (47.083%)	4.83 ± 0.21 (49.448%)
**Naproxen** 10.0 mg/kg	4.17 ± 0.30	5.73 ± 0.20 (35.395%)	5.40 ± 0.40 (43.295%)	5.20 ± 0.30 (45.205%)	4.80 ± 0.40 (56.322%)

* A statistically significant difference at the level of *p <* 0.05 in relation to the control group.

**Table 3:** COX-2 and 5-LOX enzyme inhibition results.

Compound	COX-2 Percent of Inhibition	COX-2 (IC_50_ µM)	5-LOX Percent of Inhibition	5-LOX (IC_50_ µM)
1	24.73 ± 1.11	-	34.25 ± 1.04	19.16 ± 0.96
2	18.77 ± 4.37	-	25.12 ± 1.83	39.77 ± 1.98
3	23.39 ± 0.57	-	47.02 ± 4.32	9.52 ± 1.51
4	37.71 ± 0.28	-	77.20 ± 0.26	0.30 ± 0.12
5	19.58 ± 4.55	-	46.19 ± 4.87	16.74 ± 1.79
6	23.99 ± 5.36	-	23.63 ± 2.14	-
7	24.23 ± 5.77	-	20.72 ± 4.19	-
Naproxen	72.15 ± 1.40	0.20 ± 0.02	Not tested	Not tested
Celecoxib	85.02 ± 0.31	0.07 ± 0.01	Not tested	Not tested
Zileuton	Not tested	Not tested	64.85 ± 0.39	0.36 ± 0.10

**Table 4:** The docking scores (kcal/mol) of tested compounds and positive and negative controls.

Compound	COX-2	5-LOX
1	−14.40	−7.70
2	−14.90	−7.47
3	−14.13	−6.74
4	−14.94	−8.39
5	−14.42	−7.30
6	−8.76	−7.77
7	−9.41	−7.98
Naproxen	−13.13	-
Zileuton	-	−8.77
NDGA	-	−10.67

## Data Availability

Data is contained within the article.

## Supplementary Materials

The following supporting information can be downloaded at: https://www.mdpi.com/article/10.3390/ph16050666/s1, Figure S1: ^1^H NMR spectrum (a) and ^13^C NMR (b) of compound **1**; Figure S2: ^1^H NMR spectrum (a) and ^13^C NMR (b) of compound **2**; Figure S3: ^1^H NMR spectrum (a) and ^13^C NMR (b) of compound **3**; Figure S4: ^1^H NMR spectrum (a) and ^13^C NMR (b) of compound **4**; Figure S5: ^1^H NMR spectrum (a) and ^13^C NMR (b) of compound **5**; Figure S6: ^1^H NMR spectrum (a) and ^13^C NMR (b) of compound **6**; Figure S7: ^1^H NMR spectrum (a) and ^13^C NMR (b) of compound **7**.
