# Supplementary material for: Synthesis and Investigation of Anti-Inflammatory Activity of New Thiourea Derivatives of Naproxen
Source: Pharmaceuticals (Basel). 2023 Apr 28;16(5):666. doi: 10.3390/ph16050666 (PMC10222328; doi:10.3390/ph16050666)
Supplement: Supplementary file 1 [file pharmaceuticals-16-00666-s001.zip › pharmaceuticals-2365562-supplementary.pdf]

Contents

1. NMR spectra of compounds 1-7.

^{1}H NMR spectrum (a) and ^{13}C NMR (b) of compound 1 (MN16). 3

^{1}H NMR spectrum (a) and ^{13}C NMR (b) of compound 2 (MN17). 4

^{1}H NMR spectrum (a) and ^{13}C NMR (b) of compound 3 (MN18). 5

^{1}H NMR spectrum (a) and ^{13}C NMR (b) of compound 4 (MN24). 6

^{1}H NMR spectrum (a) and ^{13}C NMR (b) of compound 5 (MN26). 7

^{1}H NMR spectrum (a) and ^{13}C NMR (b) of compound 6 (MN15). 8

^{1}H NMR spectrum (a) and ^{13}C NMR (b) of compound 7 (MN30). 9

a)

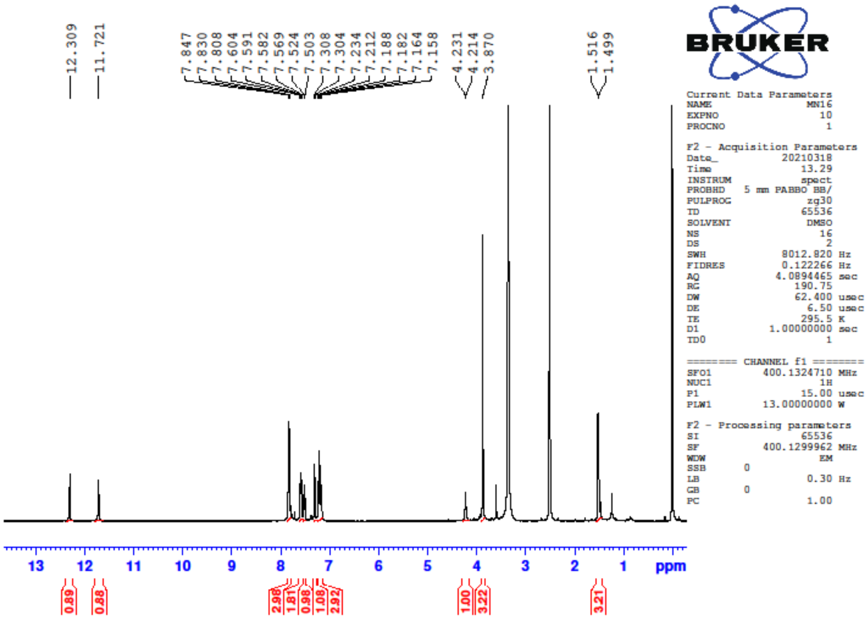

b)

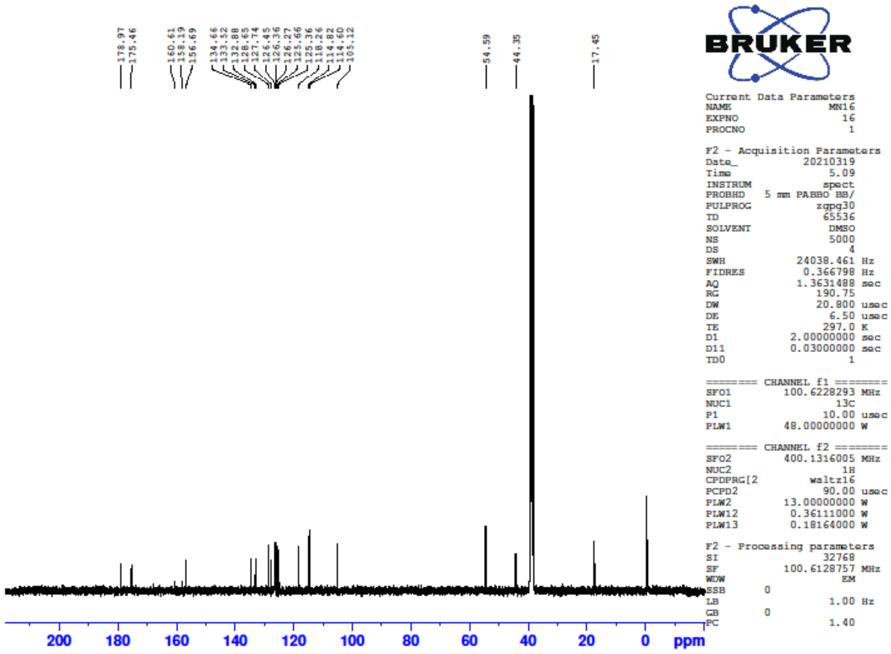

Figure S1. ^{1}H NMR spectrum (a) and ^{13}C NMR (b) of compound **1 (MN16)**.

a)

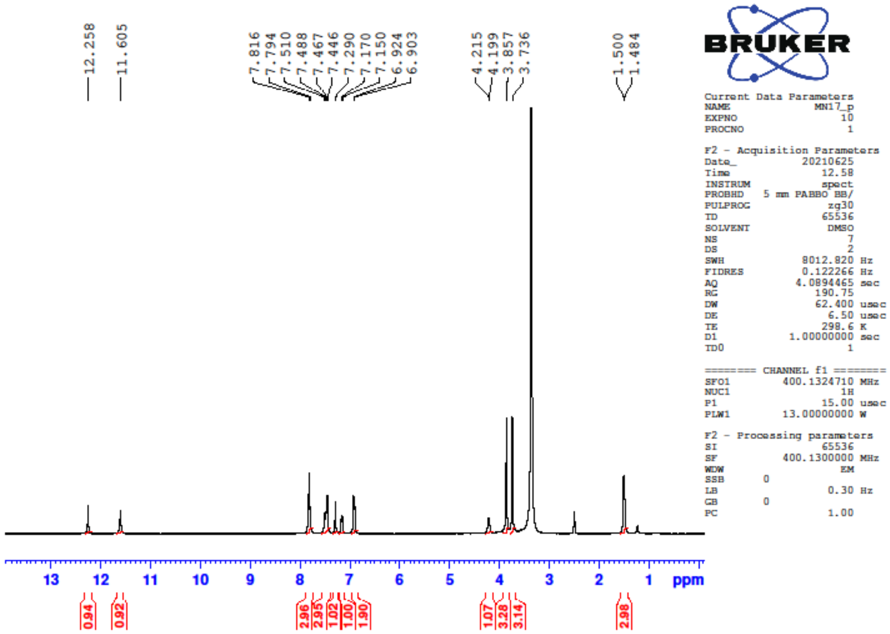

b)

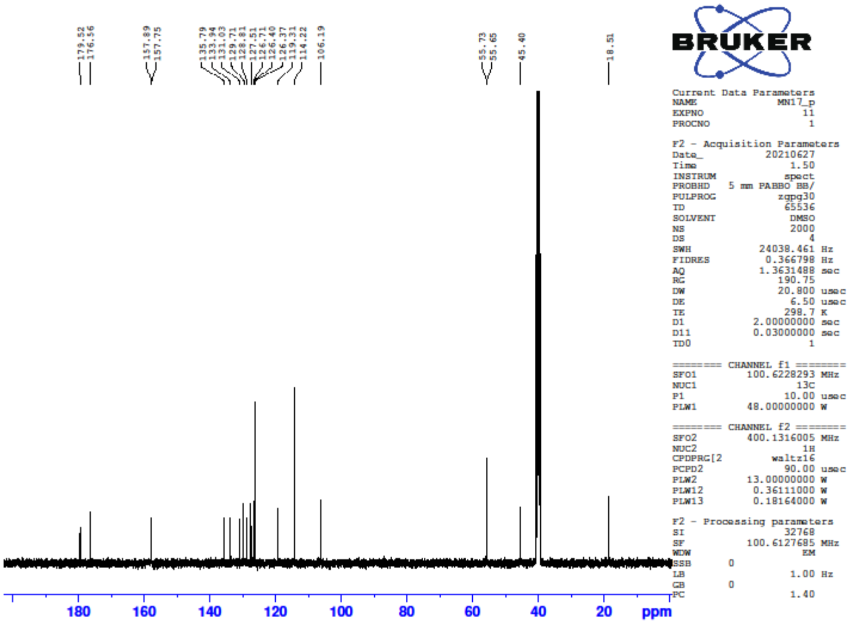

Figure S2. ^{1}H NMR spectrum (a) and ^{13}C NMR (b) of compound **2** (**MN17**).

a)

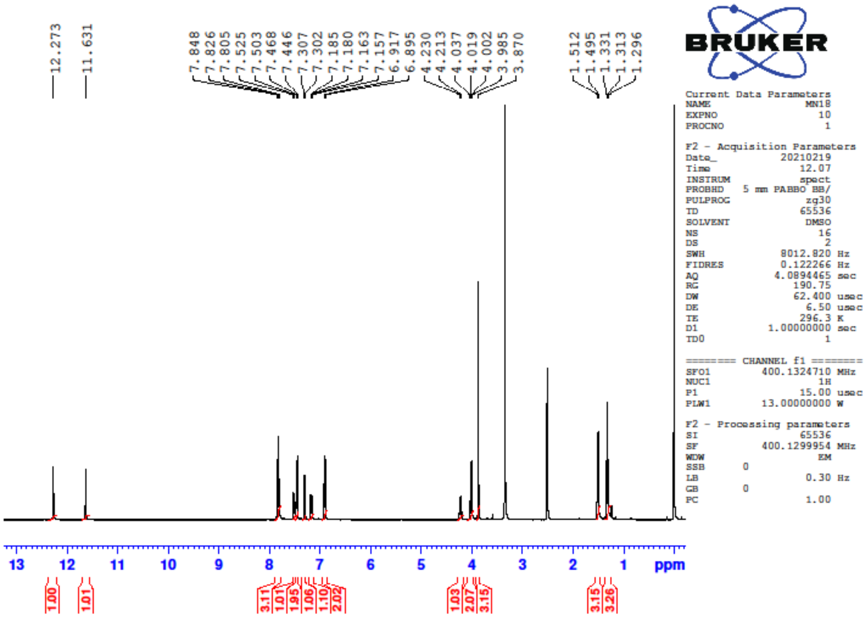

b)

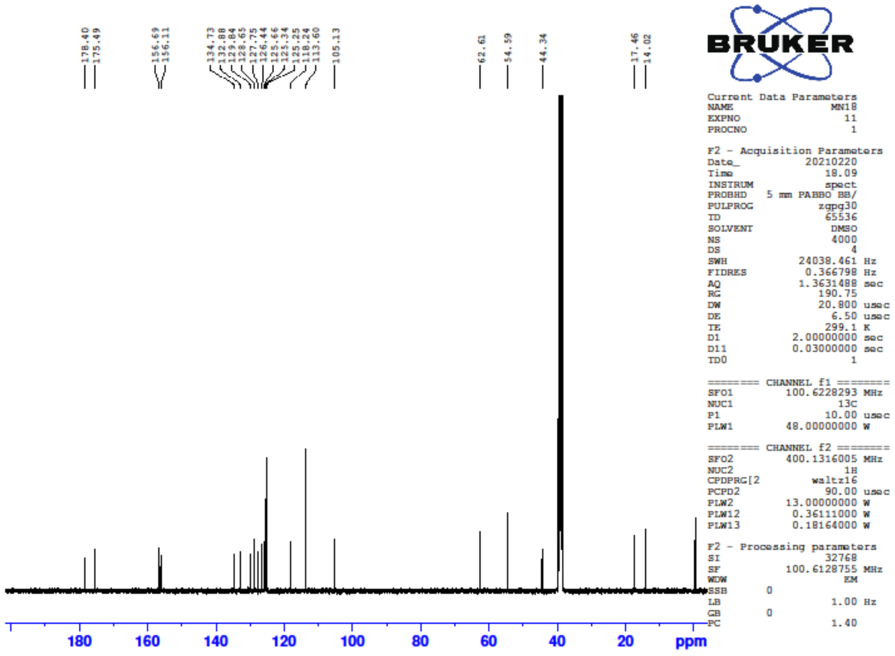

Figure S3. ^{1}H NMR spectrum (a) and ^{13}C NMR (b) of compound **3** (**MN18**).

a)

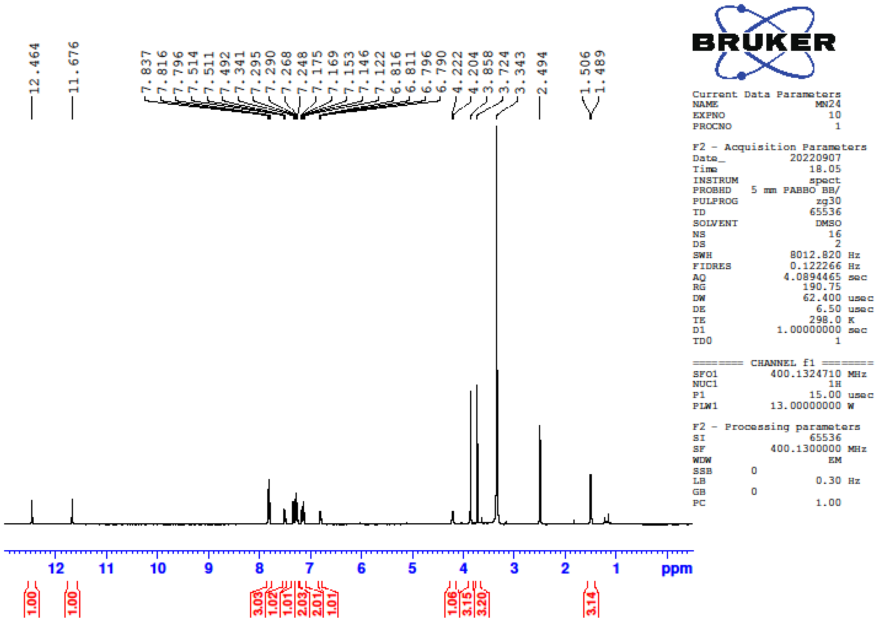

b)

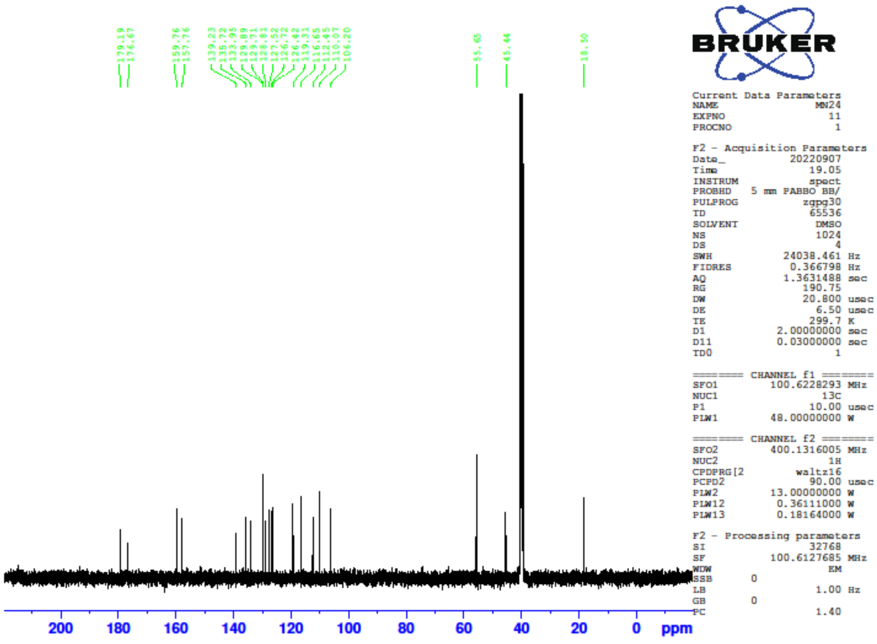

Figure S4. ^{1}H NMR spectrum (a) and ^{13}C NMR (b) of compound **4** (**MN24**).

a)

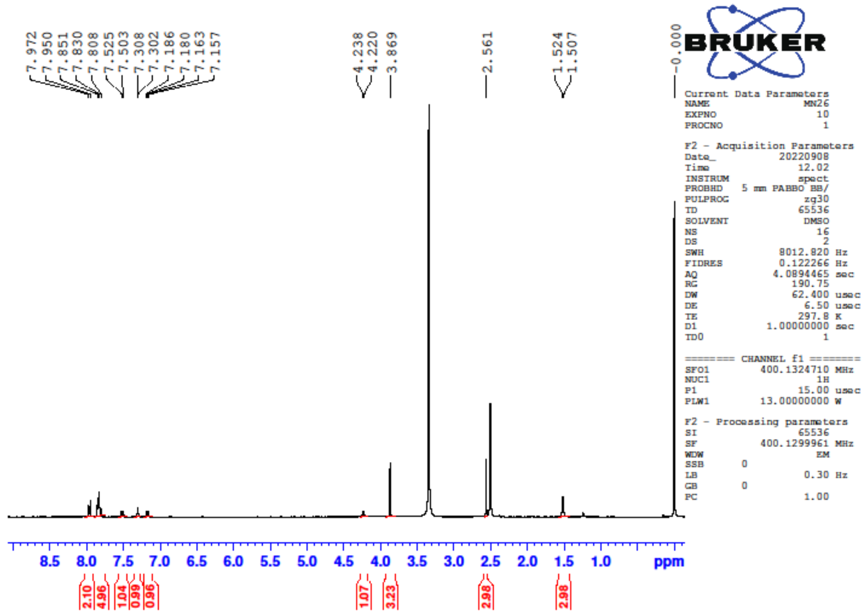

b)

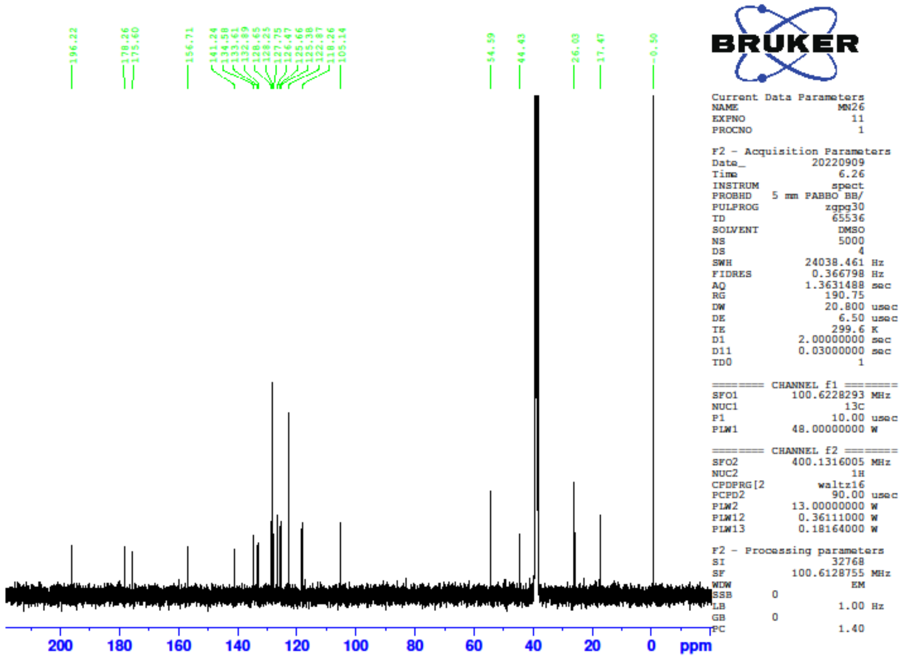

Figure S5. ^{1}H NMR spectrum (a) and ^{13}C NMR (b) of compound **5** (**MN26**).

a)

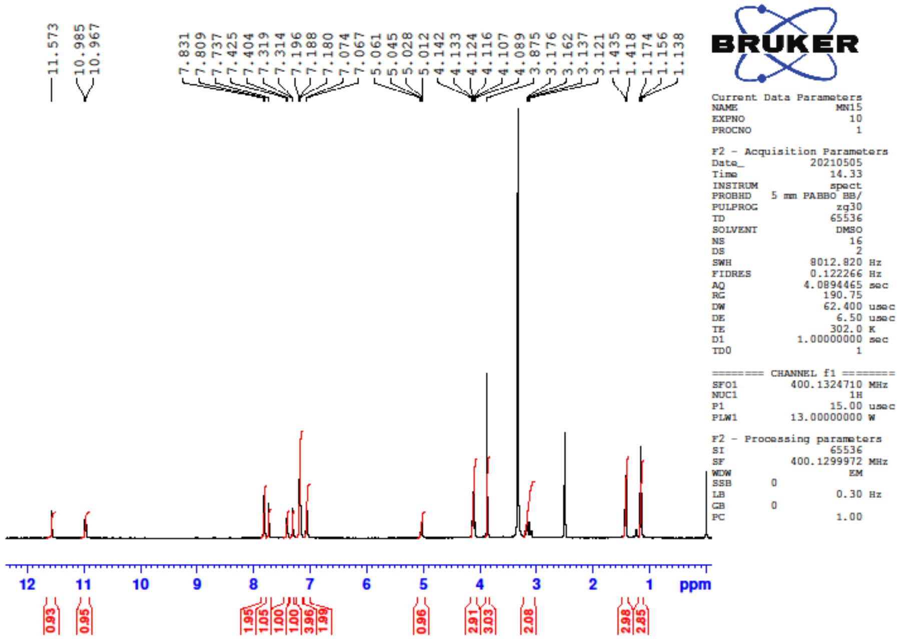

b)

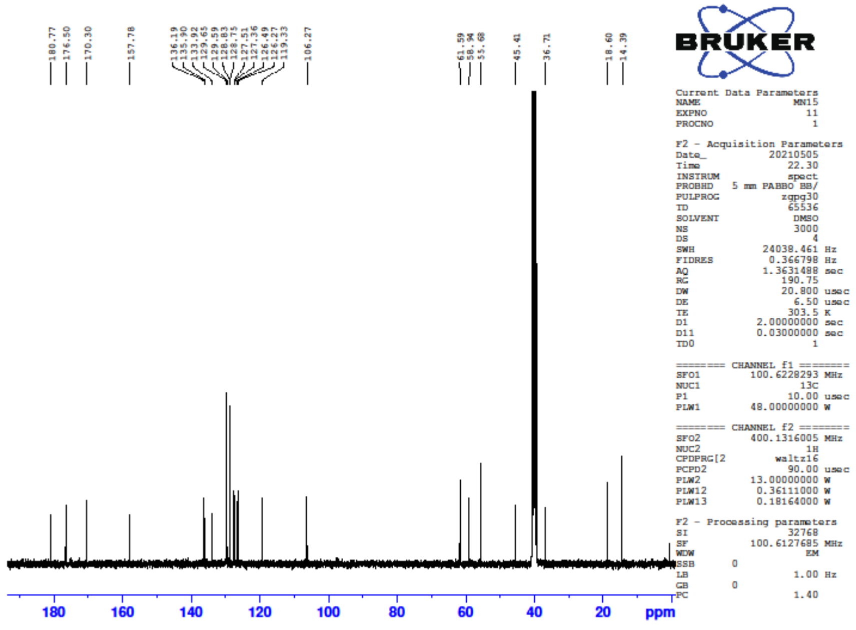

Figure S6. ^{1}H NMR spectrum (a) and ^{13}C NMR (b) of compound **6** (**MN15**).

a)

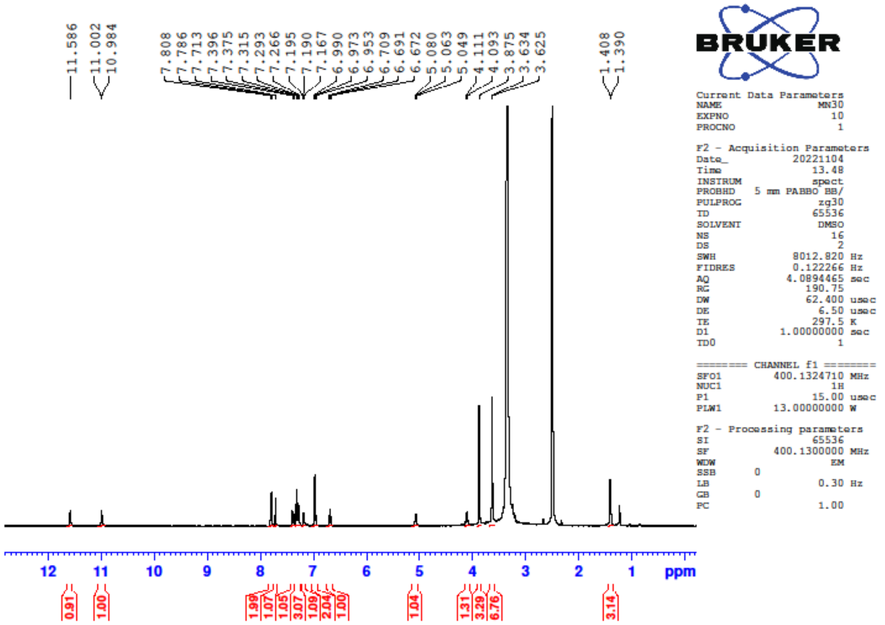

b)

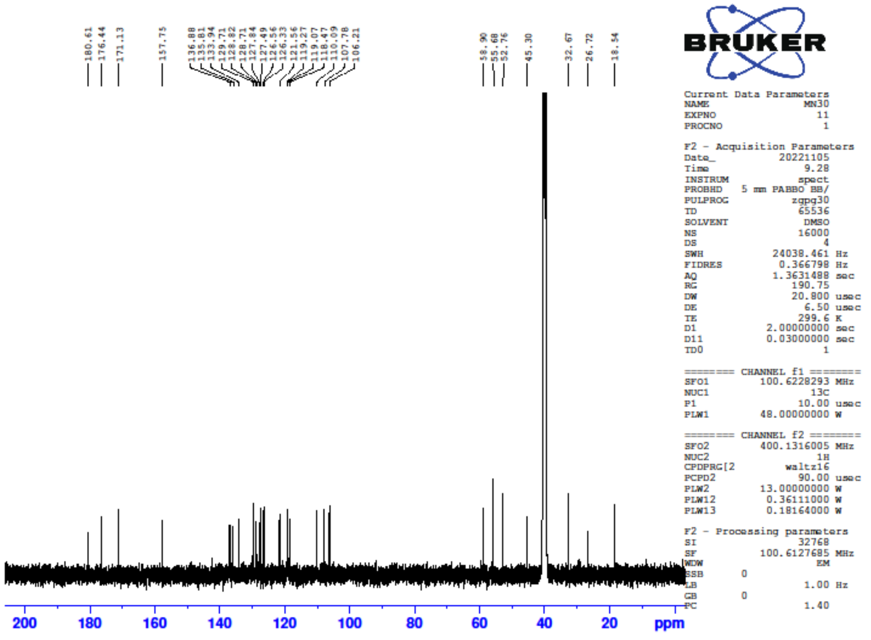

Figure S7. ^{1}H NMR spectrum (a) and ^{13}C NMR (b) of compound **7** (**MN30**).
